# Tyrosine phosphorylation unfolds nucleophosmin and disrupts its integration into the nucleolus

**DOI:** 10.1073/pnas.2533263123

**Published:** 2026-10-05

**Authors:** Rafael L. Giner-Arroyo, Adrián Velázquez-Campoy, Miguel A. De la Rosa, Irene Díaz-Moreno

**Affiliations:** ^a^ Institute for Chemical Research – Scientific Research Center “Isla de la Cartuja” (cicCartuja), University of Seville – Spanish National Research Council, Seville 41092, Spain; ^b^ Institute for Biocomputation and Physics of Complex Systems, and Department of Biochemistry and Molecular and Cell Biology, University of Zaragoza, Zaragoza 50018, Spain; ^c^Institute for Health Research of Aragón, Zaragoza 50009, Spain; ^d^Centre for Biomedical Research Network of Hepatic and Digestive Diseases, Madrid 28029, Spain

**Keywords:** nucleophosmin, tyrosine phosphorylation, liquid-liquid phase separation, protein oligomerization, nucleolus

## Abstract

Nucleophosmin (NPM1) is a multifunctional nucleolar protein essential for ribosome biogenesis, genome stability, and stress responses. Its integration into the nucleolus depends on its oligomerization and multivalent interactions that enable liquid–liquid phase separation (LLPS). Here, we investigate how tyrosine phosphorylation at Tyr17, Tyr29, and Tyr67 located within the interface between monomers at the N-terminal oligomerization domain regulates NPM1 structure and function. Replacing tyrosines with *p-*carboxymethyl-L-phenylalanine as phosphomimetic substitutions, we show that phosphorylation at Tyr17 and Tyr67 disrupts key intermonomer interactions and destabilizes the NPM1 pentameric assembly, which drives an order-to-disorder transition and impairs binding to nuclear partners. Consequently, dual phosphorylation at Tyr17 and Tyr67 disturbs both homotypic and heterotypic LLPS, thereby impairing incorporation of NPM1 into the nucleolus. This altered localization of NPM1 serves as a hallmark of p53 activation, driven both by nucleoplasmic NPM1 and by the release of ARF from NPM1-dependent sequestration in the nucleolus. Altogether, our results provide a molecular mechanistic explanation on how phosphorylation-induced structural and dynamic changes drive the release of NPM1 from the nucleolus under genotoxic stress.

## Significance

Nucleophosmin (NPM1) preserves nucleolar architecture and cellular homeostasis, yet how phosphorylation modulates its structure and function has remained unclear. We characterize a set of stress-induced tyrosine phosphorylations that collectively destabilize the NPM1 oligomer, weakening its molecular interactions and preventing incorporation into phase-separated condensates such as the nucleolus. By linking phosphorylation-driven structural remodeling to changes in subcellular localization, this work uncovers a molecular mechanism by which genotoxic stress reshapes nucleolar organization and function.

The nucleolus is a membraneless organelle (MLO) within the nucleus, known primarily as the site of ribosomal RNA (rRNA) transcription and ribosome biogenesis and assembly (1). More recently, the nucleolus has also emerged as a dynamic hub for cell cycle regulation and stress responses, coordinating various signaling pathways involved in genome stability and tumor suppression (2, 3). The structural organization of the nucleolus relies on the principles of liquid–liquid phase separation (LLPS), a process in which multivalent interactions among proteins and nucleic acids drive the formation of dense biomolecular condensates (4, 5).

Nucleophosmin (NPM1) is a multifunctional nucleolar protein that plays a central role in nucleolar assembly, ribosome biogenesis (6), centrosome duplication (7), histone chaperoning (8), and DNA damage response (DDR) via the ARF-HDM2-p53 tumor suppressor axis (9). In this DDR pathway, the nucleolar protein alternate reading frame (ARF; p19ARF in mice, p14ARF in humans) is released from sequestration by NPM1 and is relocated to the nucleoplasm, where it prevents p53 degradation upon binding to the E3 ubiquitin-protein ligase HDM2. NPM1 has likewise been reported to directly bind both to HDM2 (10) and p53 (11). Moreover, nucleus-translocated cytochrome *c* (C*c*) has also been shown to bind NPM1 in the nucleolus, competing with ARF and promoting its displacement from the organelle (12, 13).

**Fig. 1.**
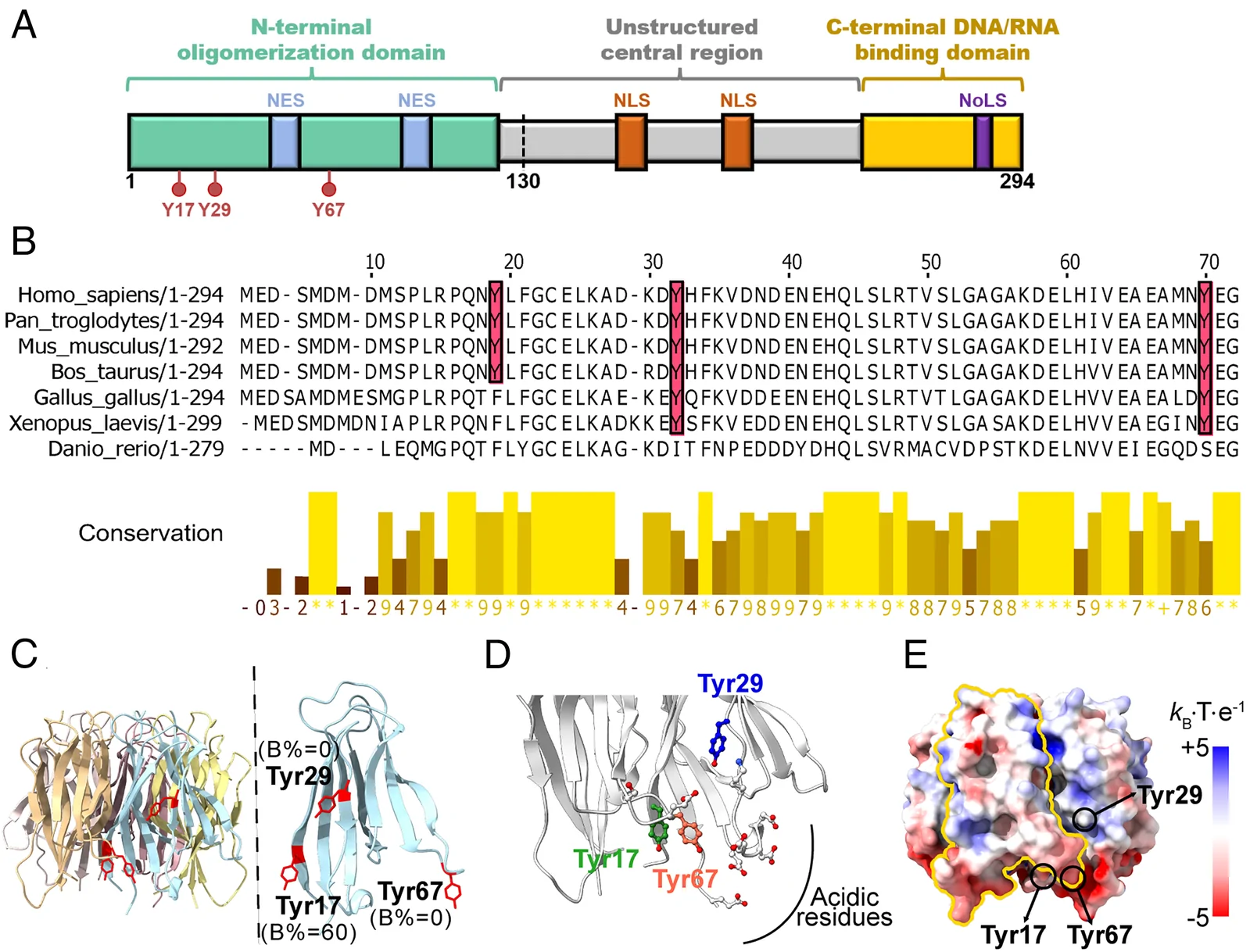
NPM1 structural organization and location of phosphorylatable tyrosines. (*A*) Domain organization of NPM1. The amino acid sequence includes an N-terminal oligomerization domain, a central unstructured region and a C-terminal DNA/RNA binding domain. The position of the three phosphorylatable tyrosine residues of interest is indicated (Y17, Y29, Y67). NES: Nuclear Export Signal. NLS: Nuclear Localization Signal. NoLS: Nucleolar Localization Signal. (*B*) Sequence alignment of the N-terminal region from different species. NPM1 sequences from representative species across various vertebrate classes were aligned using Clustal Omega (17, 18) and visualized with Jalview (19). The three tyrosine residues are highlighted in red where present. (*C*) Crystallographic structure of the oligomerization domain [PDB: 4N8M (20)]. The structure comprises a homopentamer with each monomer depicted in a different color (*Left* panel). Phosphorylatable residues Tyr17, Tyr29, and Tyr67 located in the interphase between monomers are highlighted in red in the structure of a monomer (*Right* panel). B% indicates the buried area percentage of the residue in the pentamer structure, calculated using PDBePISA (21). (*D*) Close-up view of the NPM1 region containing the three phosphorylatable tyrosine. All the charged residues in the crevice are highlighted to illustrate the disrupting effect of negative charge addition upon phosphorylation. (*E*) Electrostatic surface potential (ESP) of NPM1 core domain. Electrostatic potentials were calculated at 150 mM ionic strength. The location of phosphorylatable tyrosine surface is indicated with circles. The silhouette of one monomer is highlighted in yellow to showcase the interaction interface in the oligomer.

The NPM1 structure consists of an N-terminal oligomerization domain that forms stable homopentamers (14), a central intrinsically disordered region (IDR) rich in acidic and basic tracts, and a C-terminal nucleic acid-binding domain (NABD) (Fig. 1*A*). This charge distribution along with the multivalence conferred by the oligomerization is crucial por NPM1 integration into the nucleolus through both homotypic LLPS, driven by electrostatic complementarity of these charged tracts, and heterotypic LLPS through interactions with rRNA and proteins containing R-motifs (15, 16).

NPM1 is subject to multiple posttranslational modifications (PTMs), including acetylation (22–24), sumoylation (25, 26), ubiquitination (27, 28), and phosphorylation (7, 29–31) which act as regulatory switches for its localization and interaction dynamics. Notably, phosphorylation in residues Tyr17, Tyr29, Tyr67, and Tyr271 has been proposed to modulate NPM1 relocation from the nucleolus to the nucleoplasm in response to genomic instability (30). The dephosphorylation of these sites is mediated by the dual-specificity phosphatase DUSP3, whose loss enhances NPM1 tyrosine phosphorylation and promotes the relocalization of both NPM1 and ARF. This leads to an increase in p53 (Ser15) phosphorylation, ultimately promoting its stabilization and transcriptional activity (30). Recently, we have reported that Tyr271 phosphorylation contributes to an attenuation of the positive charge of the C-terminal nucleic acid-binding domain, interfering with its charge block-driven phase separation (32). In contrast, Tyr17, Tyr29, and Tyr67 are located within the interface between monomers at the oligomerization domain, in a region critical for oligomer stability through the establishment of intermonomer contacts (33).

In this study, we use phosphomimetic tyrosine substitutions to explore the structural and functional consequences of NPM1 phosphorylation at Tyr17, Tyr29, and Tyr67. Our findings reveal that phosphorylation in Tyr17 and Tyr67 acts synergistically to destabilize the pentameric core, induce unfolding, and hamper interaction of NPM1 with its protein partners, thereby impairing its phase separation behavior. These data highlight the impact of phosphorylation on each individual position and provide a molecular mechanistic explanation on how these modifications disrupt NPM1 integration into the nucleolus.

## Results

### Phosphorylation of Tyr17, Tyr29, and Tyr67 within the N-Terminal Oligomerization Domain Triggers NPM1 Relocation.

Among the four tyrosines recently described as phosphorylation sites involved in the regulation of NPM1 subcellular localization, residues at positions 17, 29, and 67 are located at the N-terminal oligomerization domain (Fig. 1*A*). Tyr17 is conserved among mammals, whereas Tyr29 and Tyr67 are conserved from *Xenopus* to humans (Fig. 1*B*). In the NPM1 pentameric structure, the three residues are at the interface between monomers (Fig. 1 *C* and *D*). In particular, the region containing Tyr17 and Tyr67 is especially rich in acidic residues yielding a very negative electrostatic surface potential (Fig. 1*E*), which could be subject to perturbation by repulsion in the event of negative charge addition through phosphorylation of these tyrosines.

**Fig. 2.**
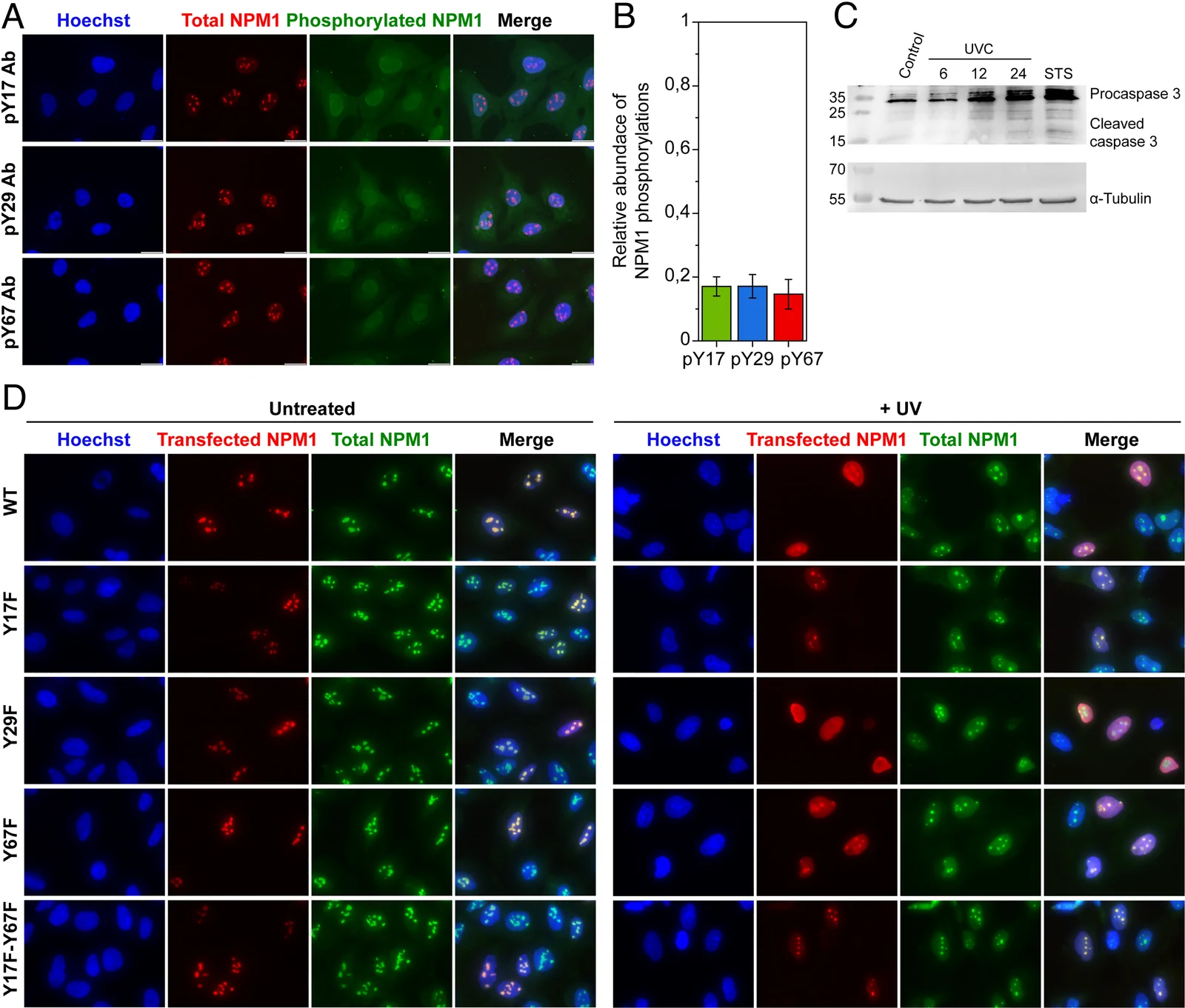
Subcellular localization of phosphorylated NPM1. (*A*) Immunofluorescence microscopy images of endogenous total NPM1 (red) and phosphorylated tyrosine 17, 29 or 67 (green) in U2OS cells. Phosphorylated species preferably show nucleo-cytoplasmic distribution. (Scale bar, 10 µm.) (*B*) Quantification of phosphorylated NPM1 fluorescence intensity ratio for the indicated antibodies (pY17, pY29, pY67). The ratio was calculated relative to the total NPM1 green fluorescence signal from an independent assay. Fluorescence signals were normalized to the concentration used for each antibody in the immunofluorescence assay. Bars represent the mean values of the distributions, error bars indicate ± one SD. (*C*) Western Blot analysis of procaspase-3 (32 kDa) and cleaved caspase-3 (17 kDa) in U2OS cells 6, 12 or 24 h following irradiation with 18 J/m^2^ UVC. Nontreated cells were used as negative control and 1 µM STS treated cells were used as positive control. α-tubulin (52 kDa) antibody was used as loading control. (*D*) Immunofluorescence microscopy images on U2OS cells of endogenous total NPM1 (green) and transfected *c*myc-tagged NPM1 Tyr-by-Phe nonphosphorylatable variants (red) before and after exposure to 18 J/m^2^ UVC. The signal from transfected NPM1 variants was detected by an anti *c*myc-tag antibody. The species unable to become phosphorylated on Tyr17 exhibit a lower tendency to nucleoplasmic redistribution upon UVC irradiation. (Scale bar, 10 µm.)

Here, we use phospho-specific antibodies designed against the synthetic peptides described by Russo et al. (30) containing phosphorylated tyrosine in positions 17, 29, and 67. Immunofluorescence assays in U2OS cells revealed detectable endogenous NPM1 pools phosphorylated at each of the three sites (Fig. 2*A*). For all three phosphorylated forms, the subcellular distribution was predominantly nucleoplasmic, in contrast to the mainly nucleolar localization observed with an antibody against total NPM1 (targeting the C-terminal domain), a signal mainly reflecting the more abundant unmodified NPM1 population. The subcellular distribution pattern of the phosphorylated forms is consistent with the results previously reported by Russo et al. (30) in MRC-5 and XPA cells, suggesting that phosphorylation-driven relocation of NPM1 may represent a general mechanism conserved across different cellular contexts. Quantification of the fluorescence signals relative to total NPM1 levels revealed similar phosphorylation levels at the three tyrosine residues (Fig. 2*B*), suggesting a shared regulatory mechanism. Caspase-3 activation was also analyzed in UV-irradiated samples (Fig. 2*C*) to assess the extent of apoptotic progression under the conditions in which these phosphorylations are detected. Caspase-3 cleavage was first detected 12 h after UV irradiation, substantially later than the 3 h and 6 h time points at which NPM1 phosphorylation events were observed (30). These results suggest that NPM1 phosphorylation events may function as early preapoptotic switches in the DNA damage response.

To assess the functional relevance of NPM1 tyrosine phosphorylation during the DDR, we generated nonphosphorylatable Tyr-to-Phe variants at the three phosphorylation sites (Fig. 2*D*). Although all variants exhibited similar subcellular localization under basal conditions, UVC irradiation resulted in increased nucleolar retention of Tyr17-deficient mutants, supporting a critical role for Tyr17 phosphorylation in NPM1 redistribution upon UV-induced DNA damage.

### NPM1 Phosphorylation at Tyr17 and Tyr67 Weakens Key Intermonomer Contacts and Promotes Loop Displacement.

**Fig. 3.**
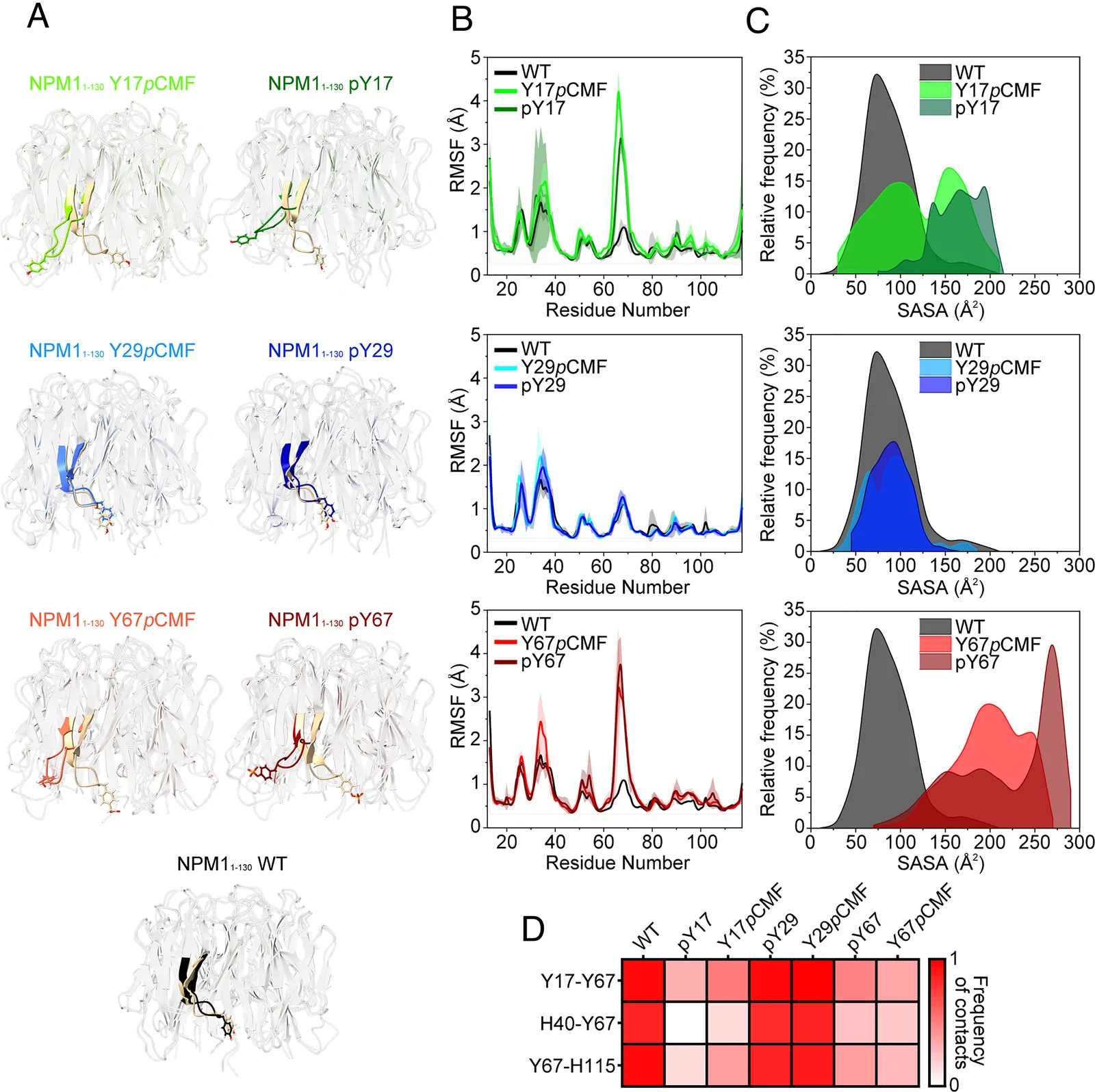
Effect of phosphorylation and phosphomimicking on the structure and dynamics of tyrosines 17, 29, and 67 in NPM1. (*A*) Ribbon representation overlay of the initial and final structures of 1-µs MDs of NPM1_1–130_ phosphorylated and phosphomimetic species. The position of the 64 to 72 residues loop and the side chain of residue 67 are highlighted in the initial (beige) and the final structures of WT (black), Y17*p*CMF (light green, *Left*), pY17 (dark green, *Right*), Y29*p*CMF (light blue, *Left*), pY29 (dark blue, *Right*), Y67*p*CMF (light red, *Left*), pY67 (dark red, *Right*). (*B*) RMS Fluctuation (RMSF) calculated from the MDs of phosphorylated and phosphomimetic NPM1_1–130_ species. The RMSF by residue of WT NPM1_1–130_ is overlapped with those of variants modified in Tyr17 (*Top* panel), Tyr29 (*Middle* panel), or Tyr67 (*Bottom* panel). Shaded areas represent the SD calculated from the three replicates. (*C*) Solvent-accessible surface area (SASA) of residue 67 of NPM1_1–130_ variants during the MDs. The relative frequency distribution of the residue in position 67 is shown for NPM1_1–130_ WT and variants modified in Tyr17 (*Top* panel), Tyr29 (*Middle* panel), or Tyr67 (*Bottom* panel). (*D*) Frequency heatmap of the contacts of key residue pairs involved in monomer–monomer interaction in MDs of NPM1_1–130_ variants. The time frequency in which the residue pairs Y17-Y67, H40-Y67, or Y67-H115 are in contact is represented for WT, phosphorylated, and phosphomimetic NPM1_1–130_. The color scale from 0 to 1 represents the fraction of the 1-µs simulation during which the residue pairs remain in contact.

As an approach to explore the structural effects of phosphorylation of these tyrosine residues, we resorted to molecular dynamics (MD) simulations on NPM1 oligomerization domain (PDB: 4N8M) variants where tyrosines were substituted with unprotonated phosphotyrosine (pTyr). As the kinases responsible for these phosphorylation events remain unknown, we employed the noncanonical amino acid *p*-carboxymethyl-L-phenylalanine (*p*CMF) as a phosphomimetic residue. *p*CMF was site-specifically incorporated using evolved tRNA-mediated genetic code expansion technology, as previously performed (32). To validate the use of *p*CMF in our in vitro experiments, MD were also performed on *p*CMF-substituted NPM1 variants. Three independent MD simulations were carried out for each system. Along 1 µs of MD simulations, the loop formed by residues 64 to 72 showed a displacement in NPM1 variants in which Tyr17 or Tyr67 were substituted by pTyr or *p*CMF residues, adopting a more external position relative to the protein mass center (Fig. 3*A*). Accordingly, average atomic RMS fluctuations (RMSF) were higher in the loop region (Fig. 3*C*) and the solvent exposure of the residue 67, as measured by the solvent accessible surface area (SASA), was increased in these variants.

Moreover, recent data have shown that the contacts between Tyr67 from one monomer and the residues His40, His115, and possibly Tyr17 from the adjacent monomer are crucial for the stability of NPM1 oligomers (33). We analyzed the frequency of native contacts between these residues over the 1-µs MD runs in the pTyr and *p*CMF variants (Fig. 3*D*). In the variants where Tyr17 or Tyr67 were substituted by either pTyr or *p*CMF, we found a decrease in the interaction frequency of residue 67 with residue 17, as well as with the two histidines in positions 40 and 115, consistent with the noticeable distancing shown in Fig. 3*A*. This effect was especially pronounced in contact with His40. No visible changes were observed in these or previous analyses upon substitution of Tyr29, suggesting that this modification exerts only a mild structural effect. Although the size and charge distribution of *p*CMF more closely resemble those of phosphotyrosine than the conventional Tyr-to-Glu substitution, we also performed MD simulations on the Y17E, Y29E, and Y67E NPM1 mutants (*SI Appendix*, Fig. S4). All Glu-substituted variants exhibited a destabilization pattern similar to that observed for the pTyr- and *p*CMF-containing proteins. In particular, substitutions at positions 17 and 67 induced loop displacement and increased solvent exposure. However, the magnitude of these structural perturbations was markedly reduced, as evidenced by the solvent accessibility analysis (*SI Appendix*, Fig. S4). These results further support the use of *p*CMF as a more faithful mimic of tyrosine phosphorylation than the conventional Tyr-to-Glu substitution.

Altogether, these observations suggest that phosphorylation of Tyr17 and Tyr67 hinders key interactions between adjacent monomers by promoting the displacement of the loop containing Tyr67. In addition, the similar behavior of pTyr and *p*CMF variants in each position validates the use of *p*CMF as a phosphomimetic residue for in vitro approaches.

### Phosphorylation of Tyr17 and Tyr67 Promotes Instability of the NPM1 Oligomeric Form.

**Fig. 4.**
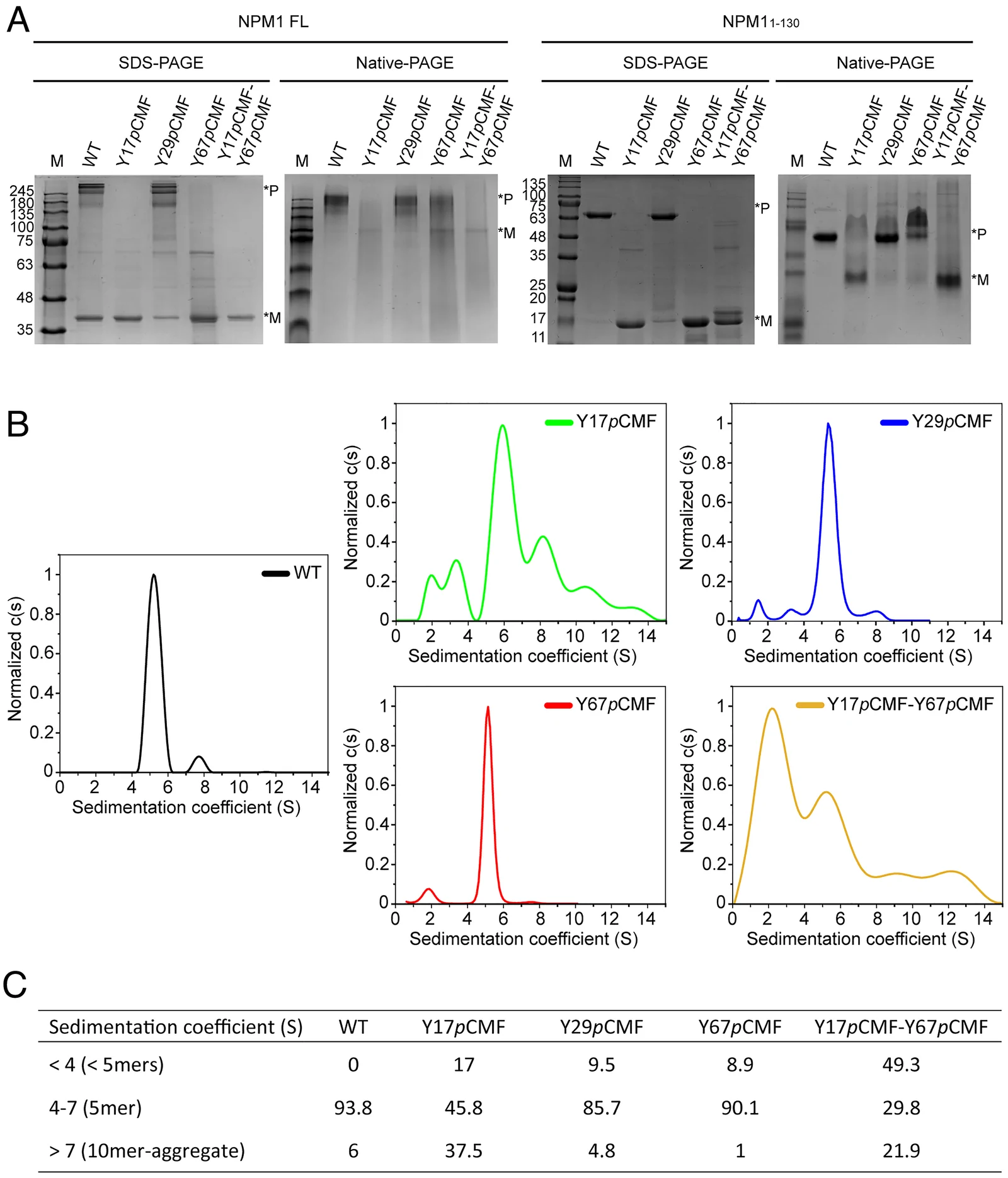
Phosphomimetic substitutions in tyrosines 17 and 67 lead to instability of NPM1 pentameric associations. (*A*) Gel electrophoresis analysis of phosphomimetic NPM1 oligomerization equilibrium. The monomer-oligomer distribution of WT and phosphomimetic single (Y17*p*CMF, Y29*p*CMF, Y67*p*CMF) and double (Y17*p*CMF-Y67*p*CMF) mutants of NPM1 FL (*Left* panels) and NPM1_1–130_ (*Right* panels) was visualized by seminative SDS-PAGE (10% SDS, no sample heating) and native-PAGE. The bands corresponding to the pentamer and monomer are indicated as *P or *M, respectively. (*B*) Velocity sedimentation AUC analysis of NPM1_1–130_ species. Normalized sedimentation coefficient distributions c(s) are shown for the WT, Y17*p*CMF, Y29*p*CMF, Y67*p*CMF, and Y17*p*CMF-Y67*p*CMF species of NPM1_1–130_. (*C*) Quantification of the percentage of oligomeric states derived from the sedimentation data in (*B*). Percentages were calculated based on the relative area under the c(s) peaks within three ranges assigned to different oligomeric states: <4S (monomeric to tetrameric species), 4 to 7S (pentameric forms), and >7S (decameric or higher-order oligomers/aggregates).

To explore whether the abovementioned phosphorylation of tyrosine residues modulates NPM1 ability to form oligomers, we generated Tyr-by-*p*CMF single mutants on positions 17, 29, and 67, as well as the double mutant on Tyr17 and Tyr67 for both the full-length (NPM1 FL) and the oligomerization domain (NPM1_1–130_) constructs. In agreement with previous data, gel electrophoresis of the wild-type (WT) sample resulted in a mix of monomers (with an apparent molecular weight of 37 kDa) and a major group of high molecular weight oligomers, even in the presence of SDS, provided the samples were not boiled (34) (*SI Appendix*, Fig. S5). The multiband pattern observed in the higher molecular weight region resembles the previously described complexity of NPM1 oligomers which, in addition to the most abundant pentameric form, includes decamers (14) and higher-order associations (35, 36). A similar monomer-oligomer equilibrium was observed for the Y29*p*CMF mutant. However, SDS–PAGE analysis of nonheated Tyr-to-Glu mutant samples failed to reveal the characteristic multiband pattern, with only monomeric and pentameric species being observed (*SI Appendix*, Fig. S6*A*). Collectively, these data suggest that *p*CMF captures key structural features of phosphotyrosine that are not fully recapitulated by the Tyr-to-Glu substitution, which may explain its limited ability to mimic the cellular localization properties of the phosphorylated protein (*SI Appendix*, Fig. S6*B*). Consistent with MDs data, the Y17*p*CMF and Y67*p*CMF single and double mutants showed a completely monomeric state (Fig. 4 *A*, *Left* panel). When the same samples were run in native conditions, the Y67*p*CMF mutant adopted a pentameric state, whereas Y17*p*CMF single and Y17*p*CMF-Y67*p*CMF double mutants appeared predominantly as a smear suggesting the occurrence of aggregation events (Fig. 4 *A*, *Left* panel). An equivalent behavior was observed in the NPM1_1–130_ oligomerization domain for all the mutants (Fig. 4 *A*, *Right* panel).

The monomer-pentamer distribution of the phosphomimetic variants in the NPM1_1–130_ construct was further assessed by analytical ultracentrifugation (AUC) on *p*CMF-containing NPM1 variants. For the WT sample, a main peak with a value between 5 and 6S corresponding to the pentamer was observed, as well as a higher size peak at 7 to 8S possibly representing the decamer (Fig. 4*B*). In agreement with the native PAGE data, Y29*p*CMF and Y67*p*CMF NPM1 variants showed a mostly pentameric organization, with a lower than 10% but detectable population of monomers. In contrast, the Y17*p*CMF and specially the Y17*p*CMF-Y67*p*CMF double mutant NPM1 variants showed a pentamer content below 50%, along with both lower associations (monomer-dimer) and higher-order particles indicative of protein aggregation (Fig. 4 *B* and *C*).

Taken together, these data indicate varying degrees of structural disruption among the three phosphorylation sites, with Tyr17 phosphorylation having the most pronounced effect—alone sufficient to destabilize the oligomeric organization, leading to monomer dissociation and aggregation. Phosphorylation at Tyr29 induces only minor structural changes, resulting in behavior closely resembling that of the WT protein. Tyr67 phosphorylation shows an intermediate effect, keeping the pentameric state as confirmed by native-PAGE and AUC but revealing a high instability of the pentamer, as inferred from its sensitivity to SDS (Fig. 4 *A*, *Left* panel). Moreover, when combined with the Tyr17 phosphomimetic mutation, the Tyr67 substitution further shifted the species toward the monomeric form, supporting a synergistic effect of the two modifications.

### Disruption of NPM1 Oligomerization by Tyr17 Phosphorylation Is Mediated Through an Order–Disorder Transition.

**Fig. 5.**
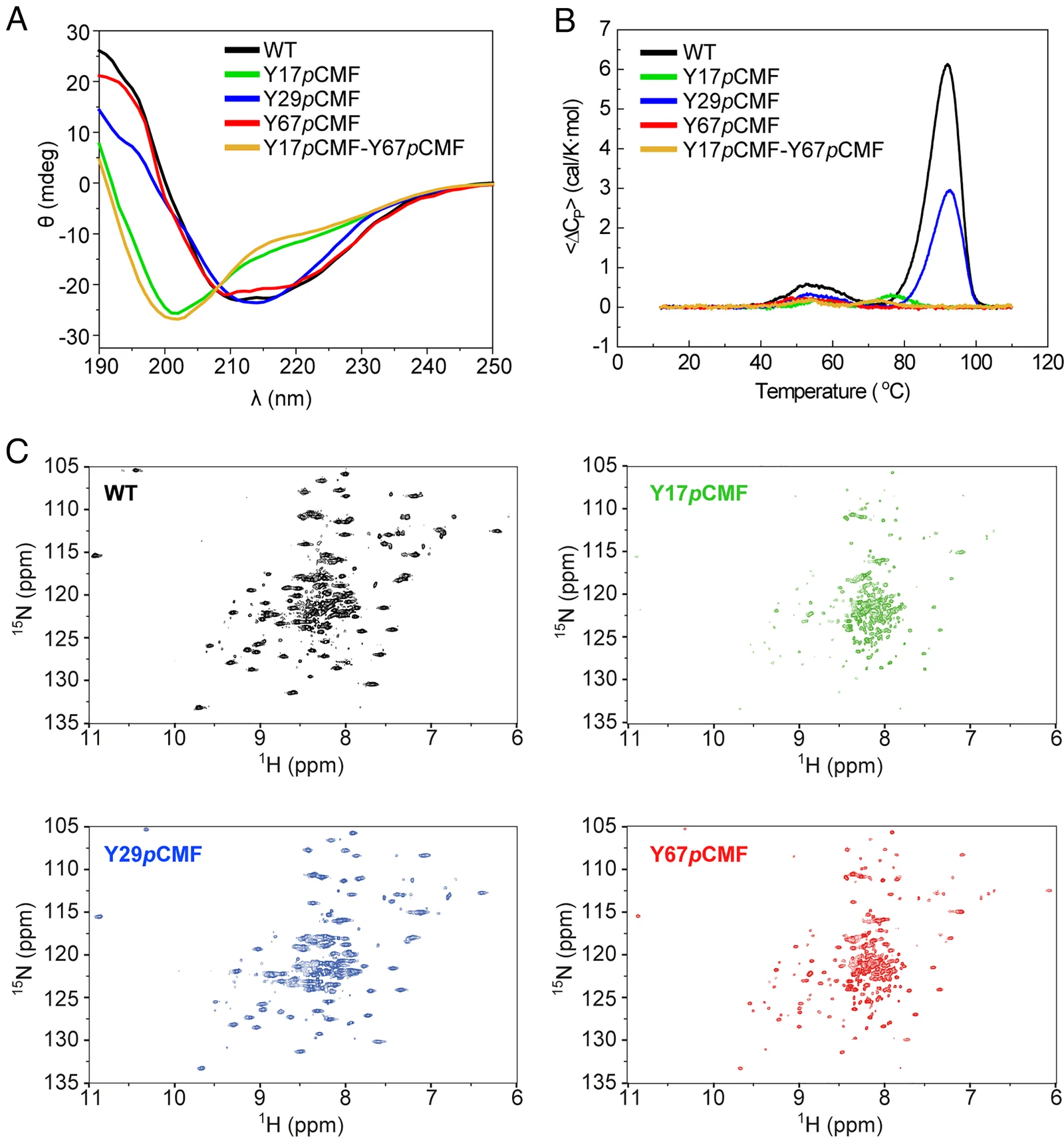
Structural and thermal stability characterization of NPM1_1–130_ phosphomimetic tyrosine mutants. (*A*) Far-UV CD analysis of the secondary structure of WT, Y17*p*CMF, Y29*p*CMF, Y67*p*CMF, and Y17*p*CMF-Y67*p*CMF NPM1_1-130_. (*B*) Differential scanning calorimetry analysis of the thermal stability of NPM1_1–130_ variants. Thermograms were analyzed with a transition and dissociation two-step model. (*C*) Two-dimensional transverse relaxation-optimized spectroscopy NMR (TROSY-NMR) spectra of ^2^H/^15^N-labeled NPM1_1–130_ species.

To delve deeper into the structural characterization of the phosphomimetic mutants, we first recorded the circular dichroism (CD) spectra of the NPM1_1-130_ mutated variants. WT, Y29*p*CMF, and Y67*p*CMF NPM1_1–130_ variants spectra confirmed their folded β-sheet secondary structure. In contrast, Y17*p*CMF, and Y17*p*CMF-Y67*p*CMF mutants exhibited CD spectra indicative of random coil content, consistent with a mostly disordered protein (Fig. 5*A*).

Differential Scanning Calorimetry (DSC) measurements of the thermal stability of NPM1 species revealed two transitions, indicating a two-step thermal unfolding process (Fig. 5*B*). For WT NPM1_1–130_, the first step is compatible with a partial unfolding of the pentamer while the second step likely corresponds to the total unfolding and dissociation of the pentamer [the trace for the second step is asymmetric, as expected for a dissociation step (37)]. The concurrence of total unfolding and oligomer dissociation in one step goes in accordance with the assembly-induced-folding mechanism proposed in Banerjee et al. (38). The first transition had an apparent T_m_ of ~53 °C, and the second one of ~92 °C. The stability of the Y29*p*CMF mutant was moderately lower, with similar T_m_ but lower enthalpy values. Conversely, the Y17*p*CMF, Y67*p*CMF, and Y17*p*CMF-Y67*p*CMF variants showed negligible denaturation enthalpies, consistent with unfolded (Y17*p*CMF and Y17*p*CMF-Y67*p*CMF) or very labile (Y67*p*CMF) NPM1 species.

These results were further confirmed by NMR spectroscopy of the WT and the three phosphomimetic single mutant NPM1_1–130_ species (Fig. 5*C*). 2D [^1^H,^15^N] TROSY spectra of deuterated WT, Y29*p*CMF, and Y67*p*CMF species exhibited disperse signals similar to the folded protein spectra previously reported (20). However, in the spectrum of the Y17*p*CMF mutant, disperse signals were faint or disappeared, with most of the backbone amide resonances collapsing at 8.0 ppm. This reflects an equilibrium between two conformations for this phosphomimetic mutant: i) a more abundant unfolded and aggregated population, and ii) a minor folded population.

### Phosphorylation of Tyr17 and Tyr67 Alters NPM1 Binding to its Partners Cytochrome *c* and ARF.

Isothermal titration calorimetry (ITC) was used to analyze the binding of C*c* to the phosphomimetic variants of NPM1_1-130_ and NPM1 FL constructs. The NPM1_1–130_ construct mainly comprises the oligomerization domain that serves as the scaffold for C*c* binding, while the NPM1 FL construct includes the IDR and NABD regions that shift from an extended state to a compact conformation upon binding (12). The unmodified variant in both NPM1 constructs exhibited two sequential binding events with C*c*, characterized by an initial high-affinity interaction (β_1_) followed by a second, weaker binding step (β_2_), indicative of negative cooperativity (Table 1 and *SI Appendix*, Figs. S7 and S8).

**Table 1.** Thermodynamic parameters for the interaction of NPM1 FL and NPM1_1-130_ variants with C*c*.

Protein complex	NPM1 variant	*β_1_*(M^−1^)	*ΔH_1_*(kcal/mol)	*β_2_*(M^−1^)	*ΔH_2_*(kcal/mol)	*ρ*	*n*
NPM1_1–130_:C*c*	WT	1.8 · 10^6^	5.3	3.7 · 10^11^	18.1	0.5	1.2
	Y17*p*CMF	2.8 · 10^4^	9.4	–	–	–	1.1
	Y29*p*CMF	2.0 · 10^6^	4.5	5.9 · 10^11^	15.8	0.6	0.8
	Y67*p*CMF	1.6 · 10^5^	9.6	–	–	–	0.8
	Y17*p*CMF- Y67*p*CMF	4.5 · 10^4^	14.0	–	–	–	0.9
NPM1 FL:C*c*	WT	1.8 · 10^6^	0.7	1.3 · 10^11^	6.4	0.2	1.1
	Y17*p*CMF	3.0 · 10^4^	9.2	–	–	–	1.1
	Y29*p*CMF	1.7 · 10^6^	1.5	1.2 · 10^11^	8.0	0.2	1.1
	Y67*p*CMF	2.3 · 10^5^	1.9	–	–	–	3.0
	Y17*p*CMF- Y67*p*CMF	–	–	–	–	–	–

Absolute errors: 0.4 kcal/mol for ∆H and 0.2 for n. Relative error: 30% for affinity constants.

Affinity constants for the first (β1) and second (β2) binding events, associated binding enthalpies (ΔH1 and ΔH2), cooperativity coefficients (ρ), and active protein fractions (n) are shown.

Phosphorylation-mimicking substitutions at Tyr17, Tyr29, or Tyr67 resulted in different levels of binding modulation. The Y17*p*CMF and Y67*p*CMF variants retained only a single binding-site with a weaker binding affinity in both NPM1 constructs, indicating that one C*c*-interacting interface was fully disrupted and the remaining site was substantially compromised. The Y29*p*CMF variant maintained the two-site binding with affinities comparable to the WT species, implying that the Tyr29 modification does not affect the formation of NPM1:C*c* adducts. Notably, the Y17*p*CMF-Y67*p*CMF double mutant in NPM_1–130_ also showed a single binding site with a lower affinity comparable to that of Y17*p*CMF alone, while no binding to C*c* was detected for the double mutant in NPM1 FL construct. Together, these data suggest that phosphorylation at Tyr17 and Tyr67 disrupts not only individual contacts but also the overall structural environment necessary for bivalent binding with C*c*.

The binding of NPM1_1–130_ phosphomimetic variants to a 37-residue peptide derived from its nucleolar partner ARF (p19ARF_1–37_) was also analyzed (Table 2 and *SI Appendix*, Fig. S9). This analysis was omitted for the NPM1 FL construct, as its titration with p19ARF_1–37_ produced nonanalyzable thermograms due to rapid molecular condensation and aggregation phenomena (20, 39) (Fig. 8). Nonetheless, p19ARF_1–37_ predominantly binds at the interfaces between monomers on the top side of NPM1 oligomerization domain (40) and, unlike when bound to C*c*, the IDR of NPM1 adopts an extended conformation when binding ARF (41), which limits the involvement of this region in the interaction. As a result, the binding differences between the two constructs are expected to be minimal.

**Table 2.** Thermodynamic parameters for the interaction of NPM1_1–130_ variants with ARF.

Protein complex	NPM1 variant	*K*(M^−1^)	*ΔH* (kcal/mol)	*k_1_*	*k_2_*
NPM1_1–130_:pARF_1–37_	WT	1.1 · 10^7^	5.1	1.1	10.0041
	Y17*p*CMF	1.8 · 10^7^	1.0	0.012	0.03
	Y29*p*CMF	1.1 · 10^7^	1.0	0.28	0.00074
	Y67*p*CMF	3.8 · 10^7^	1.9	1.5	0.011
	Y17*p*CMF- Y67*p*CMF	2.7 · 10^5^	5.1	1.7	0

Absolute error: 0.4 kcal/mol for ∆H. Relative error: 30% for affinity constants.

The global binding affinity constant (K), its associated binding enthalpy (ΔH) and the cooperativity interaction constants for nonadjacent (k1) and adjacent (k2) binding sites are shown.

The NPM1_1–130_/p19ARF_1–37_ interaction was modeled using a cooperative model reflecting the pentameric nature of NPM1, with five identical binding sites (12) and two cooperativity modifiers: k_1_ for nonadjacent sites and k_2_ for adjacent sites (*Materials and Methods*). WT NPM1_1–130_ displayed high intrinsic affinity and cooperative behavior, characterized by slightly positive nonadjacent site cooperativity (k_1_ = 1.1) and strong negative cooperativity for adjacent sites (k_2_ = 0.0041).

The Y17*p*CMF variant showed a high intrinsic affinity but greatly disrupted cooperativity, with a strong negative nonadjacent cooperativity. Consequently, while this variant maintains the ability to bind a first ligand, the binding of subsequent ligands is strongly penalized. This effect could be related to the destabilization of the NPM1 pentameric interface by the Y17*p*CMF mutation, compromising its ability to allocate the binding of multiple ligands. The Y29*p*CMF mutant preserved a similar intrinsic affinity to the WT form but displayed a modest reduction in cooperativity factors, suggesting a slightly weakened binding of multiple ligands. In contrast, the Y67*p*CMF substitution led to moderately enhanced intrinsic affinity and cooperativity. This may be attributed to the increased flexibility of the loops at the surroundings of the binding region, as observed in the MDs (Fig. 3), to facilitate the accommodation of the ARF ligands while maintaining the multiple binding sites due to the persistence of the pentameric association.

The Y17*p*CMF-Y67*p*CMF double mutant showed a clear drop in intrinsic binding affinity and a complete loss of adjacent-site cooperativity. Despite maintaining positive cooperativity at nonadjacent sites, its inability for adjacent-site binding indicates a strong impairment for the binding mechanism of multiple ligands. Moreover, the pronounced reduction in intrinsic affinity represents a qualitative shift relative to the single Y17*p*CMF and Y67*p*CMF mutants. This aligns with the marked destabilization of the pentameric structure observed when both mutations are present, which disrupts the formation of the multivalent interface required for high-affinity binding to multiple ARF ligands.

### Dual Phosphorylation at Tyr17 and Tyr67 Significantly Impairs NPM1’s Ability to Undergo Both Homotypic and Heterotypic Phase Separation.

**Fig. 6.**
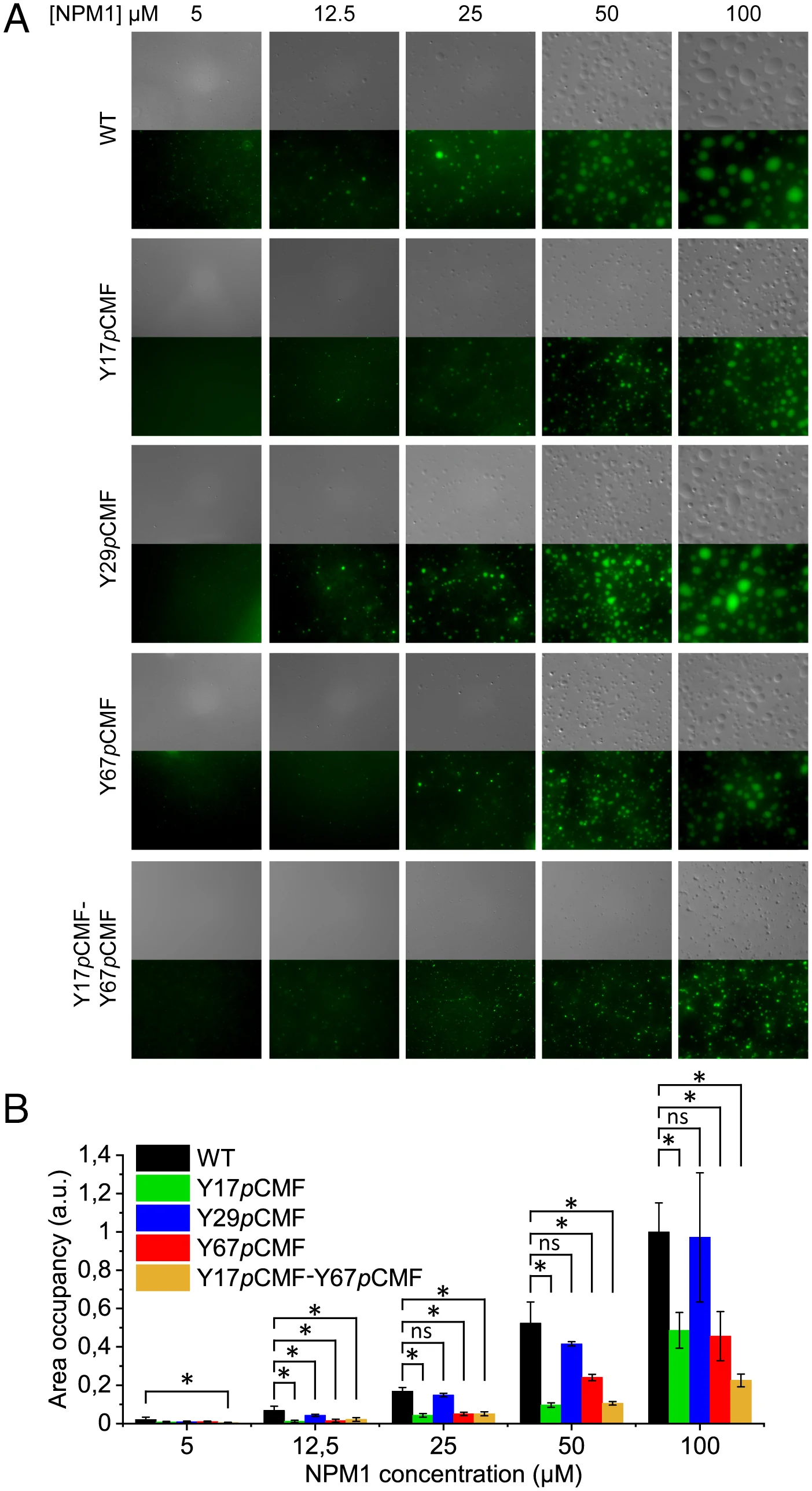
Effect of phosphomimetic substitutions on the homotypic phase separation properties of NPM1. (*A*) Homotypic liquid droplets formation at different concentrations of NPM1 phosphomimetic mutants in a buffer containing 15% Ficoll PM70 and 150 mM NaCl. For each variant, microscopy images by DIC (*Top* panel) and Oregon Green 488 fluorescence from labeled NPM1 (*Bottom* panel) are shown. (Scale bar, 10 µm.) (*B*) Quantification of the area in the microscopy fields occupied by protein condensates of WT and phosphomimetic mutants of NPM1 as a function of protein concentration. The top of the bars represents the mean values of the distributions, and the error bars indicate ± one SD. Statistically significant differences are indicated: *P*-value < 0.05 (*), not significant (ns).

The effect of phosphorylation on the ability of NPM1 FL to undergo homotypic LLPS was first examined using the branched polymer Ficoll PM70 as a crowding agent (Fig. 6), as previously reported (15). WT and Y29*p*CMF NPM1 formed liquid droplets with comparable size, morphology, and abundance across all tested molar concentrations. The Y17*p*CMF and Y67*p*CMF variants were still able to assemble condensates, albeit at a substantially higher saturation concentration (*C*_sat_) (42). This behavior is even more pronounced for the Y17*p*CMF-Y67*p*CMF double mutant, which forms liquid droplets of much smaller size and abundant only at the highest concentrations.

**Fig. 7.**
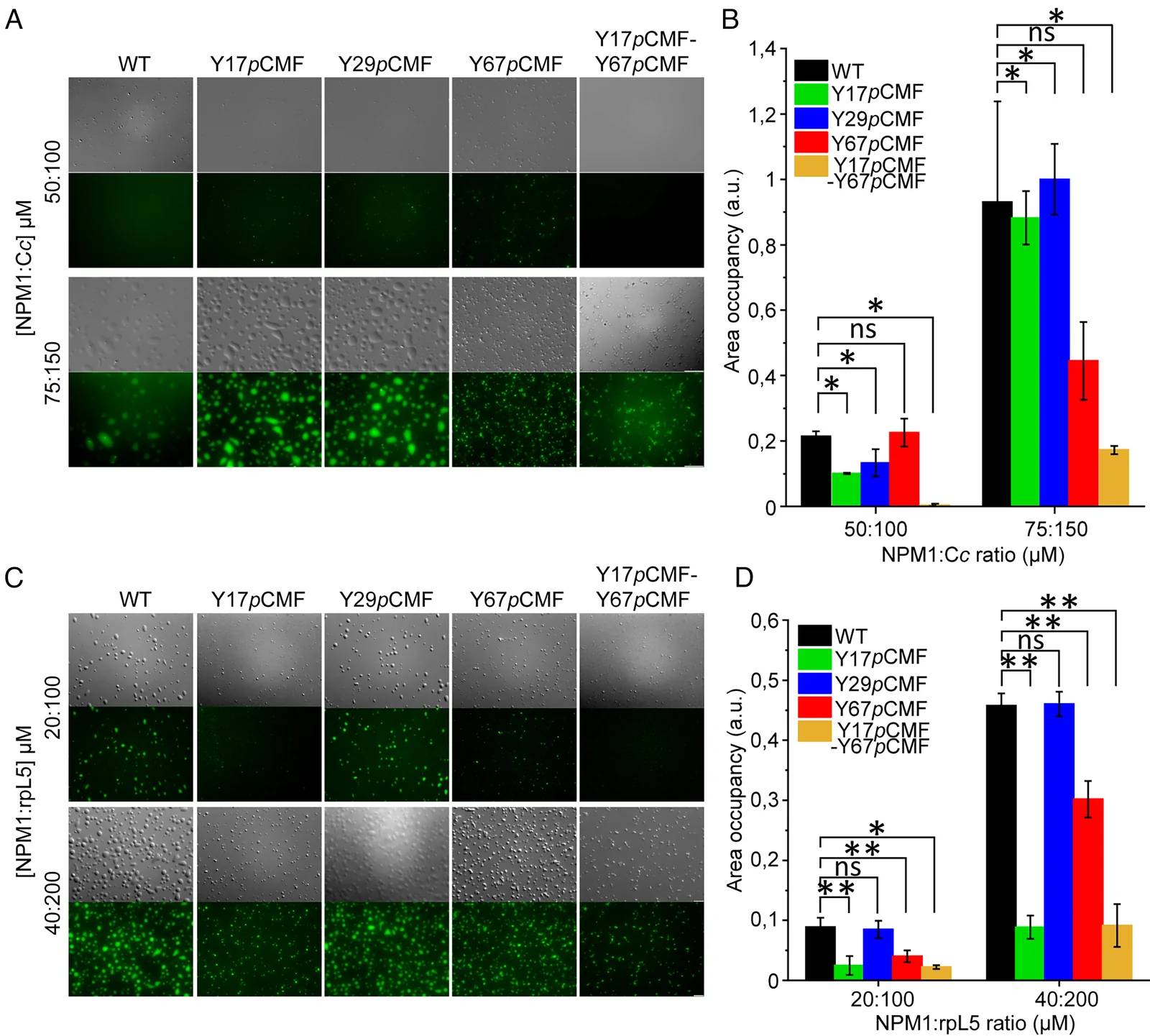
Effect of phosphomimetic substitutions on the heterotypic phase separation properties of NPM1 in the presence of C*c* or rpL5. (*A*) Heterotypic liquid droplets formation of NPM1 phosphomimetic mutants with C*c*. NPM1 was incubated with recombinant C*c* at the specified molar concentration ratios in a buffer containing 150 mM NaCl. For each variant, microscopy images by DIC (*Top* panel) and Oregon Green 488 fluorescence from labeled NPM1 (*Bottom* panel) are shown. (Scale bar, 10 µm.) (*B*) Quantification of the area in the microscopy fields occupied by protein condensates is shown for NPM1 phosphomimetic mutants and Cc at the molar concentration ratios tested. (*C*) Heterotypic liquid droplets formation of NPM1 phosphomimetic mutants with rpL5. NPM1 was incubated with synthetic rpL5 peptide at the specified molar concentration ratios in a buffer containing 150 mM NaCl. For each variant, microscopy images by DIC (*Top* panel) and Oregon Green 488 fluorescence from labeled NPM1 (*Bottom* panel) are shown. (Scale bar, 10 µm.) (*D*) Quantification of the area in the microscopy fields occupied by protein condensates is shown for NPM1 phosphomimetic mutants and rpL5 at the molar concentration ratios tested. The top of the bars represents the mean values of the distributions, and the error bars indicate ± one SD. Statistically significant differences are indicated: *P*-value < 0.05 (*), not significant (ns).

**Fig. 8.**
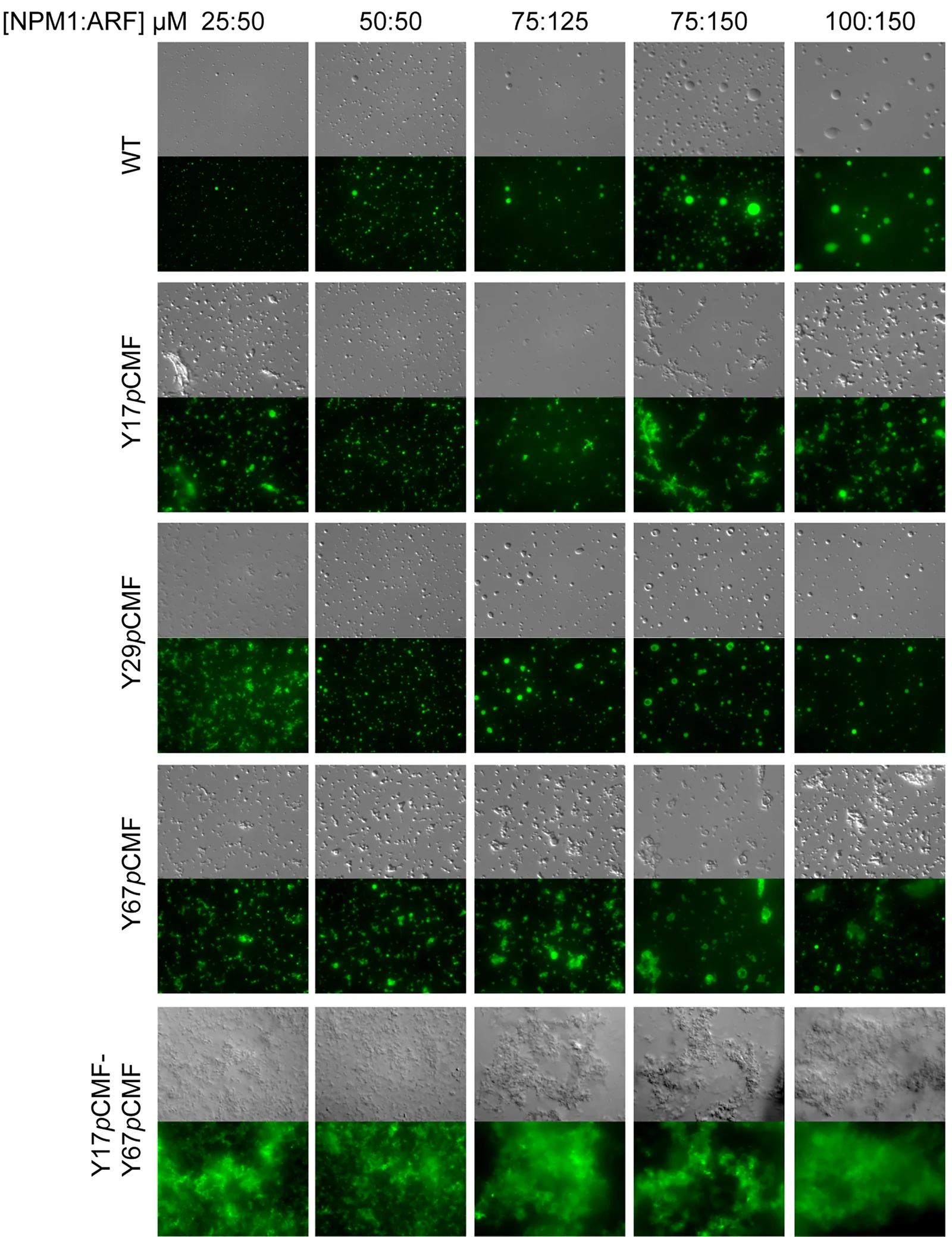
Effect of phosphomimetic substitutions on the heterotypic phase separation properties of NPM1 in the presence of ARF. NPM1 was incubated with synthetic p19ARF_(1–37)_ peptide at the specified molar concentration ratios in a buffer containing 150 mM NaCl. For each variant, microscopy images by DIC (*Top* panel) and Oregon Green 488 fluorescence from labeled NPM1 (*Bottom* panel) are shown. (Scale bar, 10 µm.)

We also explored the formation of heterotypic droplets of NPM1 FL with its known nucleolar protein partners C*c* (Fig. 7 *A* and *B*), rpL5 (Fig. 7 *C* and *D*), and ARF (Fig. 8). Titration of WT, Y17*p*CMF, and Y29*p*CMF NPM1 with C*c* yielded comparable results, producing small condensates at a 50:100 μM NPM1:C*c* ratio and larger, more wetting-prone (43) liquid droplets at the higher 75:150 μM ratio. The biomolecular condensates including C*c* and Y67*p*CMF NPM1 were similarly abundant at the lower ratio but exhibited minimal wetting and markedly slower coalescence, indicating weakened interprotein interactions and likely reduced condensate fluidity (44). The Y17*p*CMF-Y67*p*CMF double mutation resulted in a markedly reduced number of condensates, particularly at lower concentrations, while those formed at higher concentrations exhibited irregular morphologies and frequently progressed into aggregates (Fig. 7*A*). Although the presence of droplets indicates that the NPM1 double mutant retains some ability to interact with C*c*, the interaction is substantially weakened and prone to aggregation, falling below the detection threshold of ITC. In contrast, although Y17*p*CMF NPM1 exhibited reduced affinity for C*c* (Table 1), it still formed droplets comparable to those of the WT protein. This suggests that, despite lacking the intact oligomeric interface necessary for strong and well-defined C*c* binding, the single mutant retains sufficient interaction motifs to support weak, multivalent interactions to drive the formation of liquid biomolecular condensates (45). Importantly, only a single binding site for C*c* is observed in NPM1 variants, that are predominantly monomeric in solution, in contrast to the WT protein, which forms pentamers. A plausible explanation is that C*c* binding shifts the conformational equilibrium of NPM1 toward the pentameric state, incurring an energetic penalty that manifests as reduced binding affinity and lower occupancy. However, the extent of structural destabilization in the double mutant likely surpasses the threshold required to maintain the oligomeric architecture and multivalent interactions essential for phase-separated droplet formation. Likewise, the titration of NPM1 variants with the rpL5 peptide resulted in the formation of liquid droplets as previously described (46) (Fig. 7 *C* and *D*). Droplet formation was comparable between the WT and Y29*p*CMF variants but was reduced in Y67*p*CMF and further diminished in the Y17*p*CMF and Y17*p*CMF/Y67*p*CMF mutants. Consistent with the effects observed for C*c*, substitution of Tyr67 in both NPM1 variants (Y67*p*CMF and Y17*p*CMF/Y67*p*CMF) generated rpL5–NPM1 droplets with subtly altered morphology and reduced wetting behavior.

Moreover, titration of WT NPM1 with the p19ARF_(1–37)_ peptide across all tested molar ratios—previously established to favor liquid droplet formation over solid precipitates (12)—resulted in the appearance of well-defined, spherical condensates exhibiting partial wetting, as evidenced by mild spreading on the surface. The Y29*p*CMF variant displayed only moderately reduced phase separation capacity compared to WT NPM1, with condensates showing diminished coalescence at higher concentrations, slightly irregular morphologies, and a lack of wetting behavior. In contrast, the Y17*p*CMF and Y67*p*CMF mutants exhibited markedly aberrant condensate formation, producing irregular droplets even at the lowest peptide ratios and transitioning into gel/solid-like condensates or even aggregates at higher concentrations. Notably, the Y17*p*CMF–Y67*p*CMF double mutant underwent rapid and extensive aggregation upon titration with p19ARF_(1–37)_, pointing to a severe disruption of LLPS capacity when the two tyrosine residues are simultaneously modified (Fig. 8).

Notably, as previously mentioned, Tyr17 and Tyr67 are located close to the acidic tracts A1 (residues 34 to 39) and A2 (residues 120 to 130), which constitute the major region in the oligomerization domain involved in interactions with the basic tracts in the NPM1 IDR during self-assembly, as well as with the basic C*c*, rpL5, and ARF. The increased electrostatic complementarity arising from the additional negative charge in this region of NPM1 may partially compensate for oligomer instability, not only by the interaction with these protein partners but also by enhancing self-association through increased affinity for its basic tracts. This effect could contribute to the partial preservation of LLPS capacity observed in the singly modified variants. In contrast, the structural disruption caused by the double modification likely exceeds this compensatory effect and drastically disturbs its phase separation.

Altogether, these results indicate that phosphorylation at Tyr17 and/or Tyr67 markedly impairs both the homotypic and heterotypic phase separation properties of NPM1. They also reinforce the established connection between phase separation capacity and the multivalent nature of NPM1 (15, 43, 44), which is ultimately linked to the stability of its oligomeric state.

## Discussion

Our data uncover a phosphorylation-driven mechanism that controls the structural stability and nucleolar localization of NPM1. Specifically, dual phosphorylation at Tyr17 and Tyr67 synergistically disrupts the pentameric assembly. This effect stems from the fact that Tyr17 and Tyr67 are buried in the core of the pentamer, where they form key stabilizing interactions with each other as well as with His40 and His115 (33). Their modification disrupts these contacts and shifts NPM1 toward a disordered monomeric state, supporting the coupling between oligomerization and folding proposed for NPM1 (38). In this model, loss of intersubunit interactions impairs both self-assembly and folding.

Phosphorylation at Tyr17 had the strongest destabilizing effect, which was further enhanced when combined with Tyr67 modification. While Tyr67 phosphorylation alone was milder, its higher solvent-exposure (Fig. 1*C*) suggests that it might act as a priming site. MDs indicate that Tyr67 phosphorylation triggers displacement of its surrounding loop, exposing the otherwise buried Tyr17 and facilitating its access to an as-yet-unidentified kinase (Fig. 3*A* and *SI Appendix*, Fig. S10). This is in good agreement with our FoldX calculations used for rapid stability prediction (47), which identified Tyr67 phosphorylation as destabilizing and, although Tyr17 was not predicted to be destabilizing individually, highlighted the synergistic destabilizing effect of dual Tyr17 and Tyr67 phosphorylation (*SI Appendix*, Table S1). Consistently, AUC and ITC measurements also showed a markedly stronger effect for the double modification than for either single substitution, as evidenced by a further shift of the oligomerization equilibrium toward monomeric species and a reduction in the global affinity for both protein partners, respectively. Although some datasets are compatible with additive effects (for example, CD or homotypic LLPS), taken together, these observations support a cooperative destabilization when both residues are modified. This sequential phosphorylation could ultimately dismantle the pentamer.

In contrast, phosphorylation at Tyr29 had minimal impact on the NPM1 structure, oligomerization, and LLPS properties. However, the Tyr29-phosphorylated NPM1 variant localized predominantly in the nucleoplasm (Fig. 2*A*). Whether this modification affects interactions with other nucleolar components or acts in concert with Tyr17 and Tyr67 phosphorylation to modulate nucleoplasmic functions remains to be determined.

NPM1 redistribution to the nucleoplasm was observed as early as 6 h after UVC irradiation, preceding detectable caspase-3 activation by several hours (Fig. 2*C*) and supporting a role for nuclear NPM1 relocalization in the early preapoptotic stages of the DDR. Importantly, NPM1 variants impaired in Tyr17 phosphorylation showed increased nucleolar retention following UVC irradiation (Fig. 2*D*), underscoring the critical role of this phosphorylation event in mediating DDR-induced nuclear NPM1 relocalization.

Phosphorylation at Tyr17 and Tyr67 of NPM1 disrupts its pentameric assembly and compromises its multivalent architecture—an essential feature governing its ability to undergo LLPS. In the WT form, NPM1 pentamers act as a structural scaffold, enabling electrostatic contacts between acidic and basic regions, primarily located within the unstructured central region, to drive the formation of biomolecular condensates. Phosphorylation at these tyrosine residues dismantles this scaffold, effectively reducing the multivalency necessary for homotypic LLPS (5, 48).

Dual phosphorylation of NPM1 severely compromises its interaction with key nuclear partners such as p19ARF and C*c* (Tables 1 and 2). Both partners rely on the multivalent interface provided by the oligomeric NPM1 scaffold for effective binding (12, 41). These interactions play critical roles in DDR and the activation of the tumor suppressor p53 via the ARF/HDM2/p53 axis. In the nucleoplasm, NPM1 directly interacts with HDM2 (10) and p53 (11), modulating p53 stabilization. Phosphorylation-induced conformational changes hinder the ability of NPM1 to bind ARF and partially impair its capacity for LLPS. This would explain the release of phosphorylated NPM1 from the nucleolus (30), thereby allowing free ARF to bind and inhibit HDM2, block p53 ubiquitination, and promote its stabilization. Thus, phosphorylation of NPM1 may exert a dual effect—liberating ARF to inhibit HDM2, and allowing NPM1 to engage directly in DDR signaling via HDM2 and p53 interactions. Interestingly, dual phosphorylation at NPM1 results in anomalous stoichiometries of NPM1–ARF complexes (Table 2), leading to dysfunctional condensate formation characterized by irregular morphologies and aggregation-like behavior (Fig. 8). The observed aggregation may represent a phosphorylation-driven mechanism of cellular dysfunction linked to aging and disease, as seen with other LLPS-prone proteins like tau (49, 50). In vivo, NPM1 release is likely regulated through gradual, sequential phosphorylation, preventing extensive aggregation. However, we cannot rule out that such aggregation occurs in cells, potentially triggering the nuclear unfolded protein response (51) and adding an additional layer of complexity to p53 activation within NPM1-mediated DDR.

Nuclear translocation of C*c* further disrupts the NPM1–ARF interaction, promoting ARF displacement from the nucleolus (12). This competition depends on both the affinity of NPM1 for its partners and the structural integrity of its oligomeric state, which is significantly reduced by phosphomimetic substitutions at Tyr17 and Tyr67. Notably, these modifications have a more pronounced impact on condensate formation with ARF than with C*c*, albeit the binding affinity constant is higher (Tables 1 and 2 and Figs. 7 and 8). The relatively milder disruption of NPM1–C*c* phase separation suggests a potential amplification of C*c* competitive advantage, enabling it to further displace ARF from NPM1 and intensify DDR signaling.

Moreover, NPM1 also interacts and cophase separates with the 60S ribosomal subunit protein rpL5 in the nucleolus, and Tyr17 and Tyr67 constitute key residues for this NPM1:rpL5 interaction (46). Consistently, phosphomimetic substitutions at these two residues resulted in reduced liquid droplets formation. Thus, the reduced LLPS condensation with rpL5 upon phosphorylation could potentially affect the central roles of NPM1 in nucleolar architecture maintenance and ribosome maturation of pre-60S subunits (6).

Importantly, our findings build upon the work of Mitrea et al. (20), who showed that multisite phosphomimetic substitutions at serine/threonine residues shift the equilibrium of NPM1 toward disordered monomers, establishing a molecular framework for the order–disorder transition that governs NPM1 oligomerization. Their study demonstrated how interactions between charged regions are tightly regulated to control monomer assembly. Our results are fully consistent with this model and further extend it by characterizing the effect of specific tyrosine phosphorylations that have been endogenously detected in cells (30, 52–55) and supported by functional and cellular data (30), reinforcing their physiological relevance. In addition, we propose a mechanistic model in which sequential, multisite tyrosine phosphorylations act cooperatively—phosphorylation at a solvent-exposed residue, such as Tyr67, may facilitate subsequent modification of more buried sites like Tyr17—to promote unfolding and destabilization of the NPM1 oligomeric assembly. This structural rewiring compromises its ability to undergo LLPS, ultimately altering NPM1 nucleolar residence and reshaping its engagement in stress-response pathways.

Notably, the C-terminal nucleic acid–binding domain of NPM1 is also subject to phosphorylation at Tyr271 under genotoxic stress (30), along with additional modifications such as Ser254 and Ser260 phosphorylation (31). These PTMs likely disrupt NPM1 nucleolar localization by weakening its interaction with rRNA. Together, these findings suggest a synergistic regulatory mechanism in which phosphorylation events at both terminal domains cooperate to drive NPM1 delocalization outside of the nucleolus: N-terminal modifications impair multivalency-driven homotypic and heterotypic LLPS (this work), while C-terminal alterations reduce rRNA binding and interfere with charge block–driven LLPS (32). Furthermore, UV-induced genotoxic stress has been shown to trigger additional PTMs within the N-terminal oligomerization domain, including acetylation at Lys27 and Lys54—both associated with NPM1 nucleoplasmic accumulation and p53 stabilization (23). The potential crosstalk between lysine acetylation and the tyrosine phosphorylation events characterized here remains an open question, warranting further investigation into the coordinated regulation of NPM1 structure and dynamics.

In conclusion, our findings provide a comprehensive structural and functional characterization of tyrosine phosphorylations within the N-terminal oligomerization domain of NPM1, expanding upon the initial observations by Russo et al. (30). Using *p*CMF-containing phosphomimetic mutants, we define the distinct and cooperative effects of Tyr17, Tyr29, and Tyr67 modifications, uncovering a clear hierarchy in their impact on protein structure, oligomerization, and interaction capability. Among them, Tyr17 phosphorylation alone induces substantial destabilization of the pentameric assembly; Tyr67 exerts a moderate effect but enhances the accessibility and disruptive impact of Tyr17 when comodified, while Tyr29 shows minimal influence.

Strikingly, dual phosphorylation at Tyr17 and Tyr67—both located at the monomer–monomer interface—synergistically disrupts key stabilizing interactions, leading to pentamer dissociation, misfolding, and loss of multivalent binding capacity. This structural breakdown severely impairs NPM1 ability to self-associate and engage nuclear partners, essential for its incorporation into the nucleolus via phase separation. Altogether, our results establish a structural framework for understanding how individual and combined tyrosine phosphorylation events govern the dynamic redistribution of NPM1 from the nucleolus to the nucleoplasm—an early and finely tuned cellular response to genotoxic stress.

## Materials and Methods

### Cell Cultures and Treatments.

U2OS cells were cultured at 37 °C in a humidified atmosphere of 5% CO_2_. For immunofluorescence assays, cells were cultured in Dulbecco’s modified Eagle’s medium (DMEM) (Sigma-Aldrich) supplemented with 10% heat-inactivated fetal bovine serum (FBS) (Sigma-Aldrich), 2 mM L-glutamine (Sigma-Aldrich), 100 U mL^−1^ streptomycin (Sigma-Aldrich) and 100 μg mL^−1^ penicillin (Sigma-Aldrich). For the analysis of transfected NPM1 variants localization and caspase-3 activation analysis, the cell culture medium was replaced with phosphate buffer saline (PBS) and cells were irradiated with 18 J/m^2^ and changed back to culture medium. Cells were analyzed after a 6 h treatment period, unless otherwise indicated. As a positive control for caspase activation, cells were treated with 1 µM staurosporine (STS) for 6 h. Immunofluorescence and western blot assays were performed as described in *SI Appendix*, *Materials and Methods*.

### DNA Constructs.

For the recombinant expression of C*c*, a pBTR1 vector which also contained the *CYC3* gene, encoding the yeast heme lyase required for the proper folding of C*c* when expressed in bacterial cell cultures was used (56, 57).

For the recombinant expression of NPM1_1–130_ and NPM1 FL, the pET28a(+) bacterial expression vector containing the gene encoding the protein fused to an N-terminal 6xHis-tag was used (12). For the recombinant expression of the *p*CMF phosphomimetic NPM1 mutants, two bacterial expression plasmids were required: I) a pET28a(+) vector encoding an NPM1 sequence where the corresponding Tyr codon was substituted to an Amber stop codon (TAG) and II) a pEVOL vector, that encodes an unnatural tRNA/aminoacyl-tRNA synthetase (uaaRS) pair specific for the introduction of *p*CMF into the TAG codon (58). The plasmids used in this work are listed in *SI Appendix*, Table S2. The plasmids for NPM1 mutated variants were obtained by site-directed mutagenesis of pET28a(+) or pcDNA3.1, using the primers listed in *SI Appendix*, Table S3. All constructs were confirmed through Sanger sequencing (Eurofins Genomics). All oligonucleotides were purchased from Sigma-Aldrich.

### Protein Expression and Purification.

C*c* and NPM1 constructs were expressed using *Escherichia coli* BL21 (DE3) cells (70235, Novagen) and purified by cation exchange chromatography (59) or Ni-Sepharose affinity chromatography, respectively. Incorporation of *p*CMF was accomplished as previously described (58). The synthetic peptides p19ARF_(1–37)_ and rpL5 were purchased from GeneCust. The peptides were weighted and reconstituted in the appropriate buffer without any observed aggregation. C*c* protein concentration was determined by UV/Vis spectrophotometry, using an extinction coefficient of 28.92 mM^−1^ cm^−1^. NPM1 concentration was determined before every experiment with the Bradford assay (60). All NPM1 molar concentrations in this manuscript are calculated relative to monomeric form. Detailed expression and purification procedures are described in *SI Appendix*, *Materials and Methods*.

### Molecular Dynamics Simulations.

MDs were carried out on the N-terminal oligomerization domain of NPM1 (PDB ID: 4N8M) using the OpenMM toolkit, version 7.4.2 (61) and the AMBER-14SB force-field libraries (62). Structural models of phosphorylated, Tyr-to-Glu and phosphomimetic *p*CMF mutants were built using the WT unmodified protein as a template. The force field parameters of unprotonated phosphotyrosine (63) and *p*CMF (58) were used to obtain the topology and coordinate files required for the simulation with the TLEaP module of AMBER22 (64). The details of simulation parameters and analysis are provided in *SI Appendix*, *Materials and Methods*.

### CD.

CD spectra of WT and NPM1 mutants were recorded at 20 °C in the far UV region (190 to 250 nm) at 0.2 nm/min, using a 1-mm thick quartz cuvette on a J-815 CD Spectropolarimeter (Jasco), equipped with a Peltier temperature control system. Measurements were taken for 7.5 μM NPM1 FL or 15 μM NPM1_1–130_ in 10 mM sodium phosphate, 150 mM NaCl, pH 7.4 buffer. Twenty scans were averaged for each sample.

### AUC.

AUC assays were performed at the Molecular Interactions Service of the Margarita Salas Center for Biological Research (Madrid, Spain). 100 µM protein concentration of NPM1_1–130_ species in 10 mM sodium phosphate, 150 mM NaCl, pH 7.4 buffer were loaded (320 µL) into analytical ultracentrifugation cells.

### Seminative and Native Gel Electrophoresis.

For both gel electrophoresis, 0.5 µg of each pure NPM1 protein variant were prepared in 10 mM sodium phosphate, 150 mM NaCl, pH 7.4 buffer. For seminative PAGE, samples were mixed with reducing Laemmli SDS sample buffer and run without boiling in 12% SDS-PAGE gels at 200 V and RT. For native PAGE, samples were mixed with NativePAGE sample buffer (Invitrogen), loaded on 8 to 16% TGX gels (BioRad) and run at 150 V for 65 min at room temperature using a prechilled running buffer, as previously described (20). Both gels were stained with Coomassie Blue.

### DSC.

Thermal stability of NPM1 species was monitored by DSC in an Auto-PEAQ-DSC instrument (MicroCal, Malvern-Panalytical). Assays were performed in buffer 10 mM sodium phosphate, 150 mM NaCl, pH 7.4 with a protein concentration of 100 µM, scanning from 30 to 110 °C at a scanning rate of 1 °C min^−1^. Temperature unfolding data were processed by subtracting buffer–buffer scans and normalizing with the protein concentration in order to get the excess molar heat capacity of the different protein variants as a function of the temperature.

### ITC.

ITC experiments were performed in an Auto-iTC200 microcalorimeter (MicroCal, Malvern-Panalytical, Malvern, UK) at 25 °C. The experiments consisted of 19 injections of 2 μL of a reduced C*c* or p19ARF_(1–37)_ solution into a sample cell containing the NPM1_1–130_ variants, both in 10 mM sodium phosphate (pH 7.4) buffer. Protein concentrations were 100 μM NPM1_1–130_ - 200 μM C*c* or 50 μM NPM1_1–130_ - 500 μM p19ARF_(1–37)_. The injection interval was set to 150 s and the stirring speed was constantly maintained at 300 rpm. All solutions were degassed before titration. The binding isotherms were built by integrating the heat effect corresponding to each injection and normalizing by the amount of ligand injected. The binding models used in the analysis can be found in *SI Appendix*, *Materials and Methods*.

### NMR Spectroscopy.

2D ^1^H/^15^N TROSY-NMR spectra of 250 µM ^2^H-^15^N labeled NPM1_1–130_ variants in 10 mM sodium phosphate, 150 mM NaCl, 1 mM TCEP, 10% D_2_O, pH 7.0 buffer were collected at 25 °C on a Bruker Avance 700 MHz spectrometer equipped with a “QCI” quadruple-resonance cryogenic probes. NMR data were processed by using Topspin 4.3.0 and NMRPipe software. Spectra were visualized using NMRFAM-Sparky (65).

### Fluorescent Labeling.

NPM1 protein variants were labeled with a Oregon Green™ 488 maleimide probe (Molecular Probes) by taking advantage of their exposed Cys104 residue. The fluorescent probe was added to the protein solution at 10-fold molar excess from a concentrated stock solution in DMSO and the labeling reaction was performed for 2 h at RT in the dark, in 10 mM sodium phosphate, 150 mM NaCl, pH 7.4 buffer. The excess of unbound probe was removed by extensive dialysis in 10 mM sodium phosphate, 150 mM NaCl, 2 mM DTT, pH 7.4 buffer. Protein concentration and degree of labeling were determined by recording UV–visible spectra and following the indications of the manufacturer.

### Phase Separation Assays.

Homotypic NPM1 droplet formation was performed by diluting unlabeled NPM1 from a concentrated stock in 10 mM sodium phosphate, 150 mM NaCl, 2 mM DTT, pH 7.4 buffer at the indicated concentrations with a 10% of fluorescently labeled sample and adding of 15% FicollTM PM70 (Cytiva). Heterotypic NPM1-ARF, NPM1-C*c*, or NPM1-rpL5 droplet formation was achieved by mixing unlabeled NPM1 with the p19ARF_(1–37)_ and rpL5 peptides or C*c* at the indicated concentrations from concentrated stocks in 10 mM sodium phosphate, 150 mM NaCl, 2 mM DTT, pH 7.4 buffer in the presence of 10% of fluorescently labeled NPM1 sample. Extended information about the sample preparation and image acquisition is provided in *SI Appendix*, *Materials and Methods*.

## Supplementary Material

Appendix 01 (PDF)

## Data Availability

All the data are included in the article and/or supporting information.
