# Supplementary material for: Tyrosine phosphorylation unfolds nucleophosmin and disrupts its integration into the nucleolus
Source: Proc Natl Acad Sci U S A. 2026 Oct 5;123(41):e2533263123. doi: 10.1073/pnas.2533263123 (PMC13663732; doi:10.1073/pnas.2533263123)
Supplement: Supplementary file 1 [file pnas.2533263123.sapp.pdf]

Supporting Information Appendix for

Tyrosine phosphorylation unfolds nucleophosmin and disrupts its integration into the nucleolus

Rafael L. Giner-Arroyo[1], Adrián Velázquez-Campoy[2,3,4], Miguel A. De la Rosa[1], Irene Díaz-Moreno[1,*]

[1]Institute for Chemical Research – cicCartuja, University of Seville – CSIC, Seville, Spain

[2]Institute for Biocomputation and Physics of Complex Systems (BIFI), and Department of Biochemistry and Molecular and Cell Biology, University of Zaragoza, Zaragoza, Spain

[3]Instituto de Investigación Sanitaria Aragón (IIS Aragón), 50009 Zaragoza, Spain

[4]Centro de Investigación Biomédica en Red en el Área Temática de Enfermedades Hepáticas y Digestivas (CIBERehd), 28029 Madrid, Spain

[*]Corresponding author: idiazmoreno@us.es

This file includes:

SI Materials and Methods

SI References

Figures S1-S10

Tables S1-S3

SI Materials and Methods

Immunoflurescence. U2OS cells were grown for 24 h to 70% confluence in 24-well plates with 500 µL of DMEM, containing 20-mm glass coverslips. Cells were washed once in PBS and fixed for 15 min at RT in 4% paraformaldehyde (F8775-500, Sigma-Aldrich) prepared in PBS. Afterwards, cells were washed twice in PBS and permeabilized for 10 min at RT with 0.1% Triton X-100 prepared in PBS. Then, the cells were washed once with PBS and blocked in a solution of 5% bovine serum albumin (BSA) (23210, ThermoScientific) in PBS for 1 h at RT.

For NPM1 and phosphorylated NPM1 detection, coverslips were incubated with a mouse monoclonal anti-NPM1 antibody (Sigma-Aldrich; catalog number B0556; 1:500 dilution in blocking buffer) and a rabbit anti-phosphorylated NPM1 antibody (0.01 mg mL^{-1} in blocking buffer) overnight at 4 °C. Anti-phosphorylated NPM1 primary antibodies were made by ProteoGenix against the following peptides as described in Russo *et al.*[25]: p-NPM1(Y17): SPLRPQN-pY-L; p-NPM1(Y29): ADKD-pY-HFKVD; p-NPM1(Y67): AMN-pY-EGSPIK. For the simultaneous detection of endogenous NPM1 and transfected *c*myc-tagged NPM1 constructs, coverslips were incubated with a rabbit monoclonal NPM1 antibody (HPA011384, Sigma-Aldrich) and a monoclonal mouse anti-*c*myc tag antibody (05-724, Sigma-Aldrich).

Coverslips were washed three times with PBS for 5 min and probed with the secondary anti-mouse Alexa Fluor 568 (1:500) and anti-rabbit IgG-FITC antibody (1:500). Nuclei were stained by 10 min RT incubation with 200 µg/mL Hoechst dye (Sigma-Aldrich). Cells were washed with PBS and immersed in pure ethanol for 2 min, dried for a few minutes, mounted using n-propyl gallate (02370–100G, Sigma-Aldrich) and sealed with nail polish.

Samples were imaged at a Leica DM6000B (Leica Microsystems) fluorescence microscope, using a 40x objective and an ORCA-ER camera (Hamamatsu). Differential interference contrast (DIC) and fluorescence filters FITCL5 (excitation band-pass, 480/40; emission band-pass, 527/30) and Tx2 (excitation, 560/40 nm; emission, 645/75 nm) were used to visualize FITC and Alexa Fluor 568, respectively. Images were processed and analyzed with the LAS X (Leica Microsystems) and the FIJI (ImageJ) software packages.

Western Blots. For the immunoblot detection of caspase-3 and α-tubulin, cells were lysed in RIPA buffer (50 mM Tris-HCl, pH 7.4, 150 mM NaCl, 1% Triton X-100, 0.5% sodium deoxycholate and 0.1% SDS) supplemented with 1 mM Na_3VO_4, 1 mM PMSF, protease inhibitors (cOmplete Mini, EDTA-free, Roche) and 0.02 mg mL^{-1} DNase (10104159001, Roche). Cellular debris was removed by centrifugation at 21000 g for 10 min at 4 °C and 20 µg of protein was mixed with Laemmli sample buffer and resolved in 12% SDS-PAGE gels. Then, protein were transferred to a

previously activated in ethanol PVDF membrane (GE10600029, Sigma-Aldrich) and the membrane was blocked with 5% bovine serum albumin (BSA) (23210, ThermoScientific) in TTBS buffer (20 mM Tris-HCl pH 7.4, 125 mM NaCl, 0.1% Tween-20) for 1 h at RT and incubated overnight at 4 °C with the caspase-3 (sc-271028, Santa Cruz Biotechnology, 1:1000) or α-tubulin (T9026, Sigma-Aldrich, 1:500) primary antibody in TTBS. Membranes were washed 3 times with TTBS for 5 min and incubated with secondary HRP-conjugated anti-mouse IgG (A4416, Sigma-Aldrich, 1:10000) and bands were visualized using a ChemiDoc Imaging System (Bio-Rad).

Protein Expression and Purification. C*c* protein expression was achieved by culturing *E. coli* BL21 (DE3) cells (70235, Novagen) in lysogeny broth (LB) supplemented with 100 µg mL^{-1} ampicillin for 20 h at 30 °C and 180 rpm. Unlabeled $NPM1_{1\text{-}130}$ and NPM1 FL proteins were expressed in LB medium supplemented with 100 µg mL^{-1} kanamycin for 20 h at 30 °C and 180 rpm after induction at an optical density (OD600) of 0.6-0.8 by addition of 1 mM isopropyl-β-d-thiogalactopyranoside (IPTG) (R3679, iNtRON Biotechnology). Incorporation of *p*CMF was accomplished as previously described[1]. Briefly, culture was incubated at 37 °C at 200 rpm in minimal medium M9 supplemented with 100 µg mL^{-1} kanamycin and 20 µg mL^{-1} chloramphenicol. The pre-culture was pre-induced with 0.02% arabinose (A3256-25G, Sigma-Aldrich) to ensure the presence of the unnatural tRNA/aaRS pair. Protein expression was induced at an optical density (OD600) of 0.6 with 0.2% arabinose and 1 mM IPTG; *p*CMF was added after induction at a final concentration of 1 mM.

For the double isotopic labeling of $NPM1_{1\text{-}130}$ with ^{2}H and ^{15}N, *E. coli* BL21(DE3) cells were cultured for 24 h at 150 rpm and 30 °C in 1 L of M9 medium, supplemented with 50 µg mL^{-1} kanamycin and 20 µg mL^{-1} chloramphenicol for *p*CMF variants, 0.2% ^{2}H-^{12}C-glucose, 0.1% $^{15}NH_4Cl$ and 99.8% ^{2}H-D_2O.

The synthetic peptides $p19ARF_{(1–37)}$ and rpL5 were purchased from GeneCust. The peptides were weighted and reconstituted in the appropriate buffer without any observed aggregation.

After protein expression, cells were collected at 5,000 g for 10 min and resuspended in 40 mL of lysis buffer composed of 20 mM Tris-HCl, 0.8 M NaCl, 10 mM imidazole, 1 mM DTT, pH 8.0 (for cell expressing NPM1 species) or 10 mM Tricine-NaOH pH 8.5 (for cells expressing C*c*) supplemented in both cases with 1 mM phenylmethylsulfonyl fluoride (PMSF), protease inhibitors (cOmplete Mini, EDTA-free; Roche), 0.2 mg mL^{-1} lysozyme and 0.02 mg mL^{-1} DNase. Cell suspensions were ruptured by sonication (cycles of 30 s at 35 % of amplitude, 60 s of rest, 4 min total time, on ice) and centrifuged at 20000 g, 1 h to remove cell debris.

Protein purification of all NPM1 constructs was achieved by affinity chromatography using a Ni Sepharose 6 Fast Flow column (GE Healthcare) previously equilibrated with lysis buffer. NPM1 protein was eluted using an imidazole gradient from 0 to 300 mM. The protein-containing fractions were dialyzed against 10 mM sodium phosphate buffer with 150 mM NaCl pH 7.4 (or without NaCl for ITC experiments) and concentrated in Amicon® Ultra-15 Centrifugal Filter Units (10 or 30 kDa cutoff) until reaching the required protein concentration. NPM1 concentration was determined before every experiment with the Bradford assay[2], using the Bio-Rad Protein Assay Dye Reagent (5000006, Bio-Rad) with BSA (23210, ThermoScientific) as a standard. All NPM1 molar concentrations in this manuscript are calculated relative to monomeric form.

C*c* purification was performed as previously reported through cation exchange chromatography[3]. Fractions containing pure C*c* were dialyzed against 10 mM sodium phosphate, pH 7.4, and concentrated in an Amicon® Ultra-15 Centrifugal Filter Unit (3 kDa cutoff). Protein concentration was determined by UV/Vis spectrophotometry, using an extinction coefficient of 28.92 mM^{-1} cm^{-1}.

Mass Spectrometry Analysis. Mass spectrometry (MS) assays were performed at the biomolecular mass, proteomics, and metabolomics (BIO-MS) laboratory at the University Pablo de Olavide (UPO). Acrylamide gel bands were destained with ammonium bicarbonate and acetonitrile. DTT and iodoacetamide were respectively used to break disulphide bonds and carbamidomethylate cysteine residues. Samples were incubated overnight at 37 °C with bovine trypsin at ratio 1:10 (enzyme:substrate). After extraction with acetonitrile and acidification, samples were desalted and concentrated using C18-filled tips. To test the incorporation of the non-canonical amino acid *p*CMF, MS spectra were analyzed with an ORBITRAP Q-Exactive Plus or a MALDI-TOF Ultraflextreme system (Bruker) configured in positive reflectron mode. For each spectrum, results of 3000 laser shots were averaged.

Molecular Dynamics simulations. Simulations were performed under periodic boundary conditions, using an orthorhombic cell geometry and particle mesh Ewald (PME) electrostatics, with an Ewald summation cut off of 8 Å. The system was neutralized with sodium and chlorine counter-ions according with the total charge of the protein and solvated using an optimal 3-charge, 4-point rigid water model (OPC) molecules[4]. The whole system was subjected to 5000 steps of energy minimization at 298 K. Temperature was controled by using a Langevin thermostat[5] with a friction coefficient of 1 ps^{-1} and a step size of 0.001 ps. For each NPM1 species, three replicas beginning at different coordinates were subjected to 1 µs of MD simulation.

CPPTRAJ module of AMBER was used for the trajectory analysis[6]. Molecular graphics were depicted with UCSF ChimeraX software[7]. Contacts were calculated with Contact Map Explorer tool of MDTraj library[8]. Cutoff distance was set at 3.5 Å. Solvent-accessible surface areas (SASA) were calculated with SASA Calculation tool of MDTraj library.

Analytical Ultracentrifugation. Analytical Ultracentrifugation (AUC) experiments were carried out at 20 °C and 48000 rpm in a XL-A analytical ultracentrifuge (Beckman-Coulter Inc.) equipped with UV-VIS absorbance detection systems, using an An-50Ti rotor, and 12 mm Epon-charcoal standard double-sector centerpieces. Sedimentation profiles were recorded at 280 nm. Differential sedimentation coefficient distributions were calculated by least-squares boundary modelling of sedimentation velocity data using the continuous distribution c(s) Lamm equation model as implemented by SEDFIT[9]. Experimental *s* values were corrected to standard conditions (water, 20 °C, and infinite dilution) using the program SEDNTERP[10] to get the corresponding standard *s* values ($s_{20,w}$).

FoldX ΔΔG calculation. The effect of tyrosine phosphorylation on NPM1 stability was estimated using FoldX 4[11]. The crystal structure of $NPM1_{1\text{-}130}$ (PDB: 4N8M) was pre-processed with the RepairPDB tool to optimize side-chain conformations. Phosphorylated variants were then generated using the BuildModel tool. The change in free energy (ΔΔG) was obtained as the difference between the ΔG of each modified variant and that of the WT species. Positive ΔΔG values indicate destabilization, whereas negative values indicate an increased protein stability.

Isothermal Titration Calorimetry Analysis. The binding isotherms were analyzed applying binding models, depending on the interaction under study, employing non-linear least-squares regression analysis with user-defined fitting routines in Origin 7.0 (OriginLab). For the binding of $NPM1_{1\text{-}130}$ and C*c*, an interaction model considering two cooperative ligand binding sites was applied for estimating binding affinity constants (β_1 and β_2), enthalpies (ΔH_1 and ΔH_2), cooperativity factors (ρ) and apparent fractions of active (binding competent) protein (n)[12,13].

For the binding of $NPM1_{1\text{-}130}$ and $p19ARF_{(1\text{–}37)}$, a model considering a pentameric protein with five identical and cooperative ligand binding sites was applied for estimating affinity constants and cooperative interaction constants, with an intrinsic binding affinity K and cooperativity factors k_1 and k_2 modulating the effective binding affinities during progressive site occupancy within the pentamer. Accordingly, ligand binding occurs sequentially, with the first ligand binding with an intrinsic affinity K and subsequent ligands binding with modified affinities: the second with Kk_1 (non-adjacent site) or Kk_1k_2 (adjacent site), the third with Kk_1k_2 or $Kk_1k_2^2$ (central site), the fourth with Kk_1k_2, and the fifth with $Kk_1k_2^2$ [14].

Phase Separation Assays. In all cases, 2 µL of the sample mixtures were mounted in sealed chambers, consisting of a glass slide and coverslip and kept together by a layer of perforated 3M^{TM} double-sided, 100-µm thick tape[15,16]. Samples were equilibrated for 15 min at RT and four microscopy field images from two mounted samples were acquired for each experimental condition using a Leica DM6000B fluorescence microscope (Leica Microsystems), with a 40× objective and an ORCA-ER camera (Hamamatsu). Differential interference contrast (DIC) and fluorescence filter FITCL5 (excitation band-pass, 480/40; emission band-pass, 527/30) were used to visualize Oregon GreenTM 488. Image processing was performed with the LASX (Leica Microsystems) and FIJI (ImageJ) softwares[17]. FIJI's trainable Weka segmentation tool was employed for image segmentation and droplet area occupancy quantification[18]. Pairwise comparisons of droplet area occupancy were done using a Mann-Whitney U test in Origin 2020b (OriginLab Corp.) software.

SI References

1. Guerra-Castellano, A. *et al*. Mimicking tyrosine phosphorylation in human cytochrome *c* by the evolved tRNA synthetase technique. *Chem. Eur. J.* 21, 15004–15012 (2015).
2. Bradford, M. M. A rapid and sensitive method for the quantitation of microgram quantities of protein utilizing the principle of protein-dye binding. *Anal. Biochem.* 72, 248–254 (1976).
3. Gomila, A. M. *et al*. Phosphorylation disrupts long-distance electron transport in cytochrome *c*. *Nat. Commun.* 13, 7100 (2022).
4. Izadi, S., Anandakrishnan, R. & Onufriev, A. V. Building water models: a different approach. *J. Phys. Chem. Lett.* 5, 3863–3871 (2014).
5. Andersen, H. C. Molecular dynamics simulations at constant pressure and/or temperature. *J. Chem. Phys.* 72, 2384–2393 (1980).
6. Roe, D. R. & Cheatham, T. E. III. PTRAJ and CPPTRAJ: software for processing and analysis of molecular dynamics trajectory data. *J. Chem. Theory Comput.* 9, 3084–3095 (2013).
7. Meng, E. C. *et al*. UCSF ChimeraX: tools for structure building and analysis. *Protein Sci.* 32, e4792 (2023).
8. McGibbon, R. T. *et al*. MDTraj: a modern open library for the analysis of molecular dynamics trajectories. *Biophys. J.* 109, 1528–1532 (2015).

9. Schuck, P. Size-distribution analysis of macromolecules by sedimentation velocity ultracentrifugation and Lamm equation modeling. *Biophys. J.* 78, 1606–1619 (2000).
10. Laue, T. M., Shah, B., Ridgeway, T. M. & Pelletier, S. L. Computer-aided interpretation of analytical sedimentation data for proteins. In *Analytical Ultracentrifugation in Biochemistry and Polymer Science* (eds Harding, S. E., Rowe, A. J. & Horton, J. C.) 90–125 (Royal Society of Chemistry, 1992).
11. Schymkowitz, J. *et al.* The FoldX web server: an online force field. *Nucleic Acids Res*. 33, W382–W388 (2005).
12. Freire, E., Schön A. & Velazquez-Campoy, A. Isothermal titration calorimetry: general formalism using binding polynomials. *Methods Enzymol.* 455:127-155 (2009).
13. Vega, S., Abian, O. & Velazquez-Campoy, A. A unified framework based on the binding polynomial for characterizing biological systems by isothermal titration calorimetry. *Methods* 76:99-115 (2015).
14. Taneva, S.G. *et al*. A mechanism for histone chaperoning activity of nucleoplasmin: thermodynamic and structural models. *J. Mol. Biol*. 393:448-463 (2009).
15. Casado-Combreras, M. Á. *et al*. Cytochrome *c* prompts the recruitment of its nuclear partners SET/TAF-Iβ and NPM1 into biomolecular condensates. *iScience* 27, 108593 (2024).
16. Casado-Combreras, M. Á., De la Rosa, M. Á. & Díaz-Moreno, I. Protocols for monitoring condensate formation and dynamics between the phase-separating proteins SET/TAF-Iβ and cytochrome *c*. *STAR Protoc.* 6, 103796 (2025).
17. Schneider, C., Rasband, W. & Eliceiri, K. NIH Image to ImageJ: 25 years of image analysis. *Nat. Methods.* 9, 671–675 (2012).
18. Arganda-Carreras, I. *et al*. Trainable Weka Segmentation: a machine learning tool for microscopy pixel classification. *Bioinformatics* 33, 2424-2426 (2017).

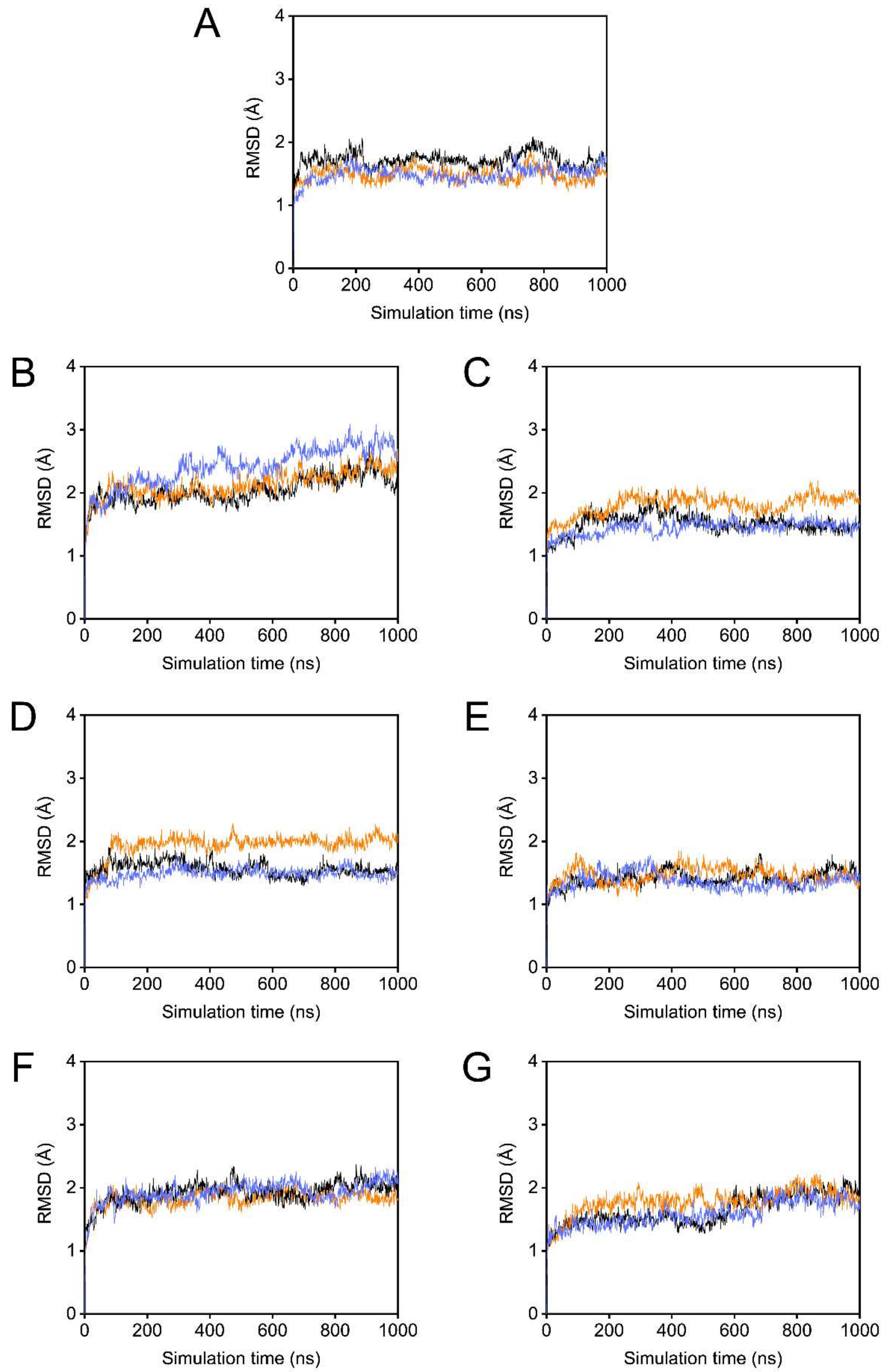

Figure S1. Root mean square deviation (RMSD) of the three MD simulations replicates of NPM1$_{1-130}$ WT **(A)**, pY17 **(B)**, Y17*p*CMF **(C)**, pY29 **(D)**, Y29*p*CMF **(E)**, pY67 **(F)** and Y67*p*CMF **(G)**.

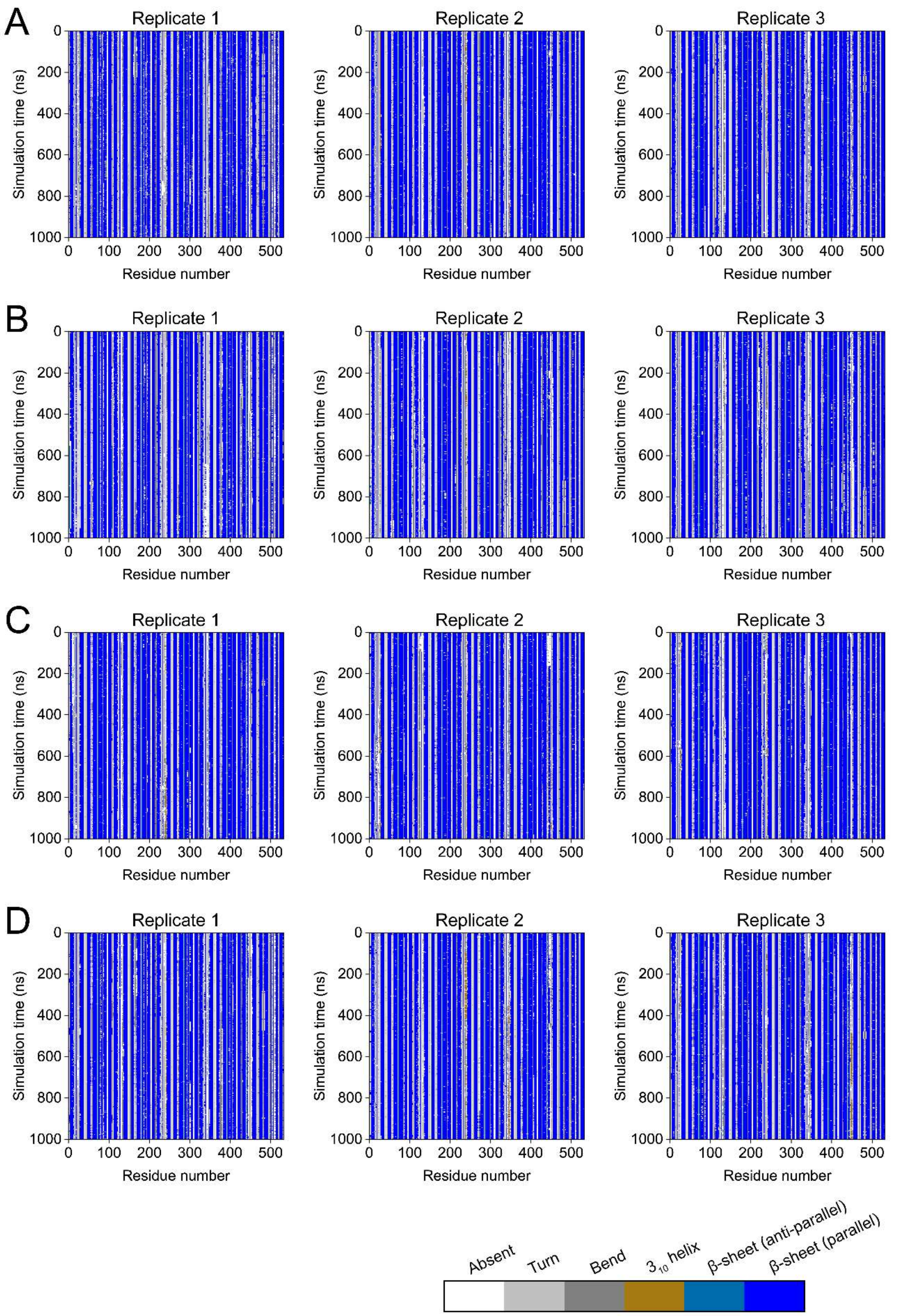

Figure S2. Secondary structure Kabsch plots of the three MD simulations replicates of NPM1$_{1\text{-}130}$ WT **(A)**, pY17 **(B)**, pY29 **(C)** and pY67 **(D)**.

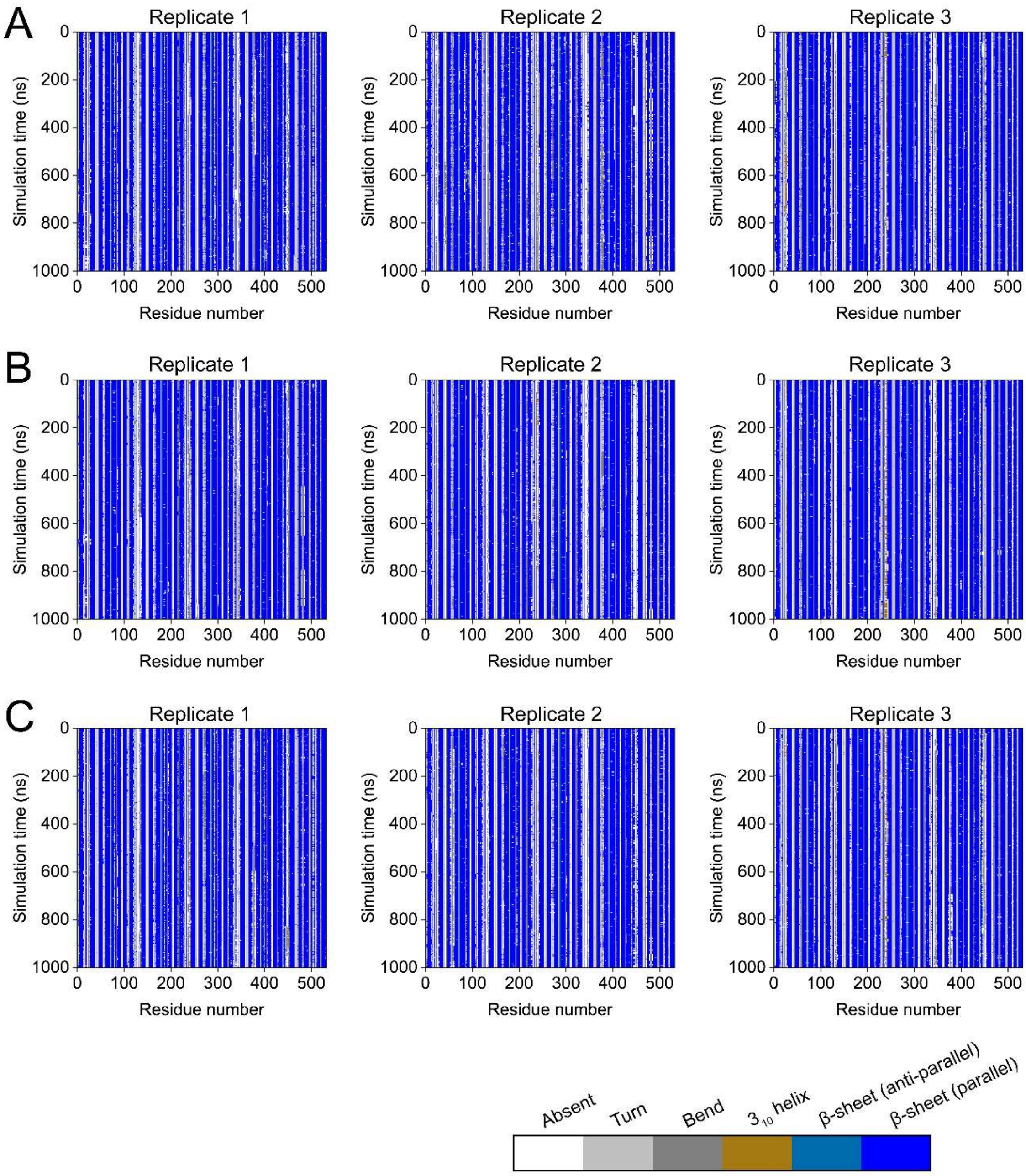

Figure S3. Secondary structure Kabsch plots of the three MD simulations replicates of $NPM1_{1\text{-}130}$ Y17*p*CMF **(A)**, Y29*p*CMF **(B)** and Y67*p*CMF **(C)**.

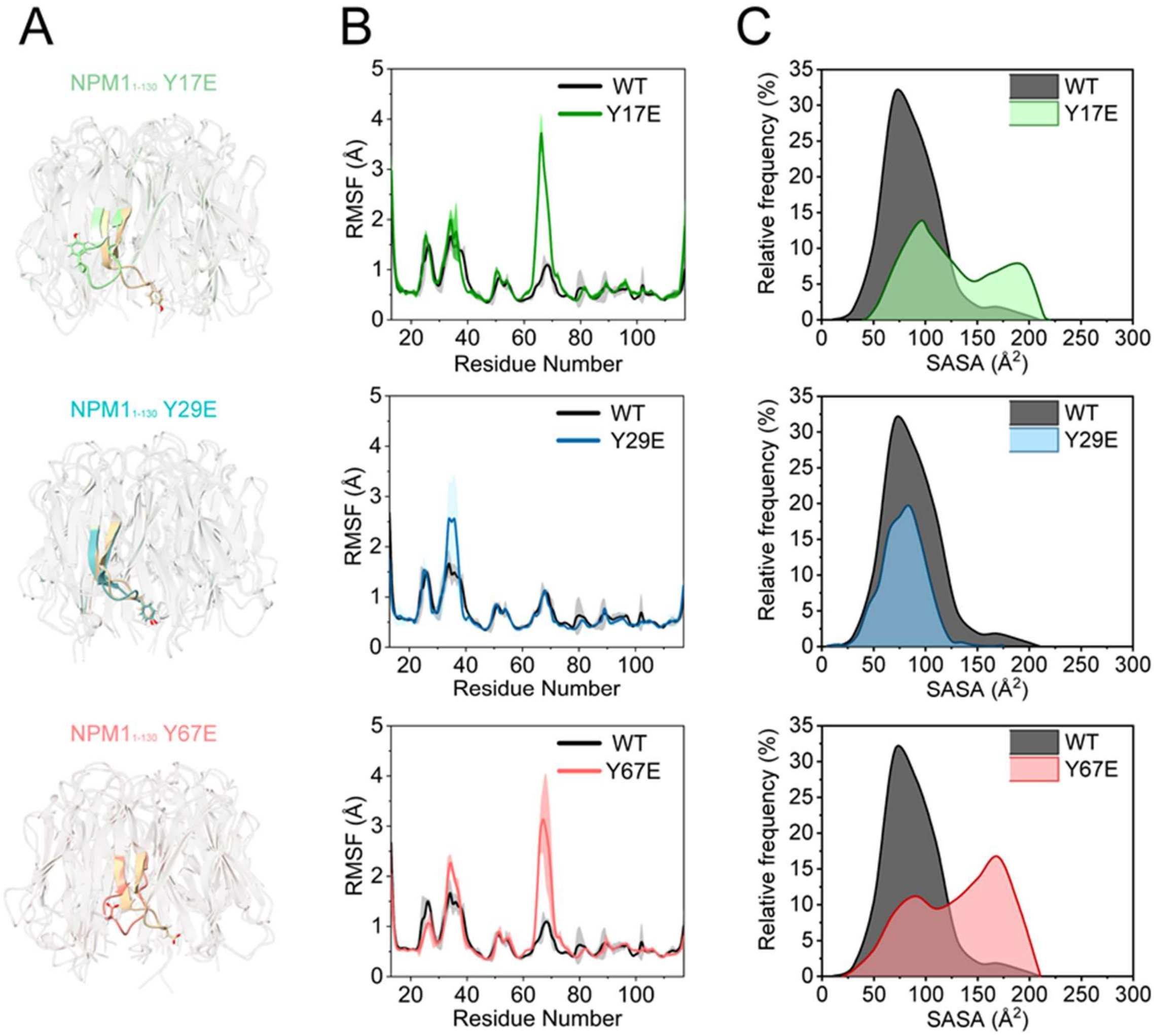

Figure S4. MD simulations of NPM1 Tyr-by-Glu mutant variants. A) Ribbon representation overlay of the initial and final structures of 1-µs MDs of $NPM1_{1\text{-}130}$ Tyr-by-Glu substituted variants. The position of the 64-72 residues loop and the side chain of residue 67 are highlighted in the initial (beige) and the final structures of Y17E (green), Y29E (blue) and Y67E (pink). **B)** Root Mean Square Fluctuation (RMSF) calculated from the MDs of Y17E (top panel), Y29E (middle panel) and Y67E (bottom panel) $NPM1_{1\text{-}130}$ species. The RMSF by residue of WT $NPM1_{1\text{-}130}$ is overlapped with those of the modified variants. Shaded areas represent the standard deviations calculated from three replicates. **C)** Solvent-accessible surface area (SASA) of residue 67 of $NPM1_{1\text{-}130}$ variants averaged for the three MD replicates. The relative frequency distribution of the residue in position 67 is shown for $NPM1_{1\text{-}130}$ WT and Y17E (top panel), Y29E (middle panel) or Y67E (bottom panel).

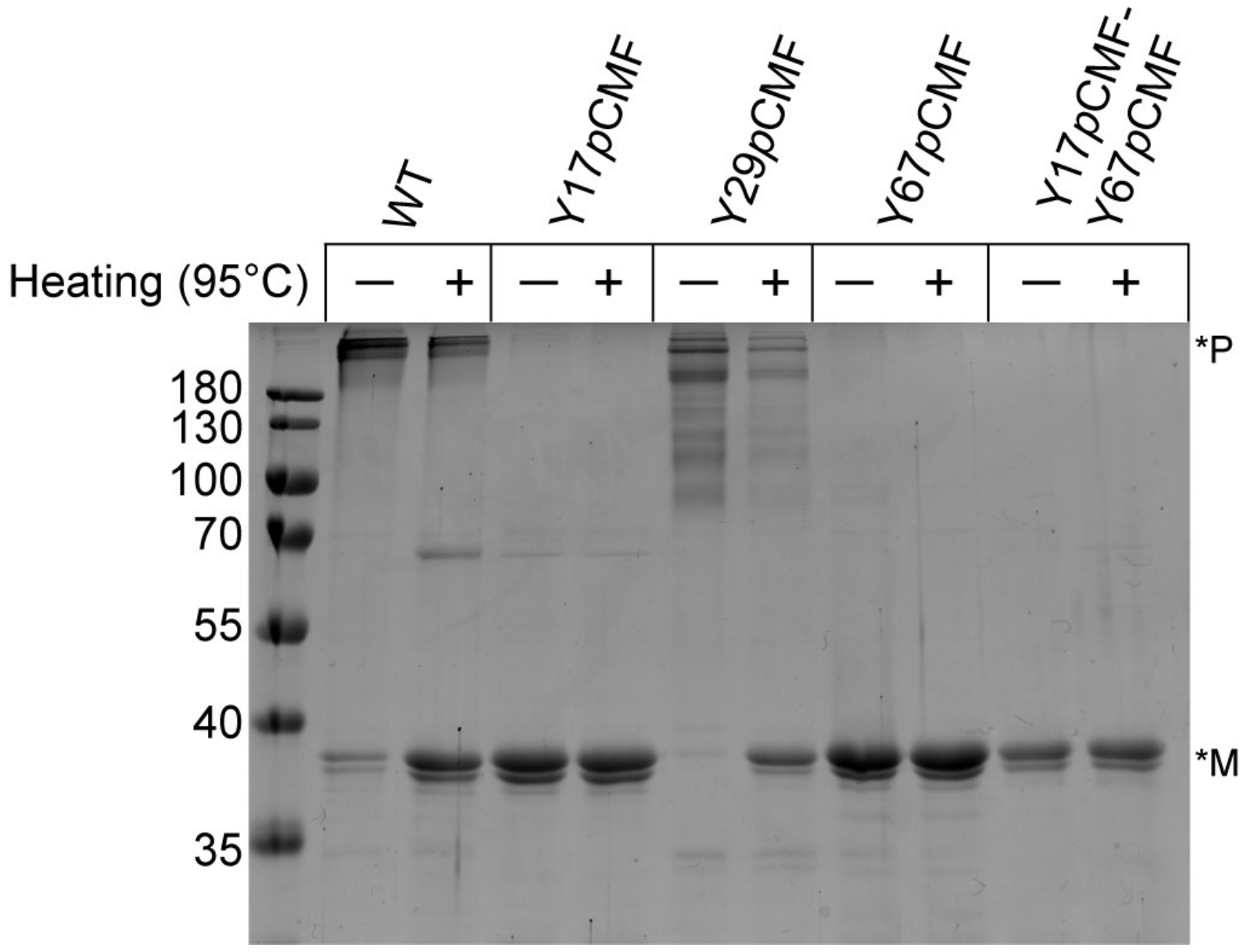

Figure S5. Gel electrophoresis analysis of phosphomimetic NPM1 oligomerization equilibrium upon sample boiling. The SDS-PAGE shows a side-by-side comparison of the monomer-oligomer distribution in heated and unheated samples of WT and phosphomimetic single (Y17*p*CMF, Y29*p*CMF, Y67*p*CMF) and double (Y17*p*CMF-Y67*p*CMF) mutants of NPM1 FL. The bands corresponding to the pentamer and monomer are indicated as *P or *M, respectively.

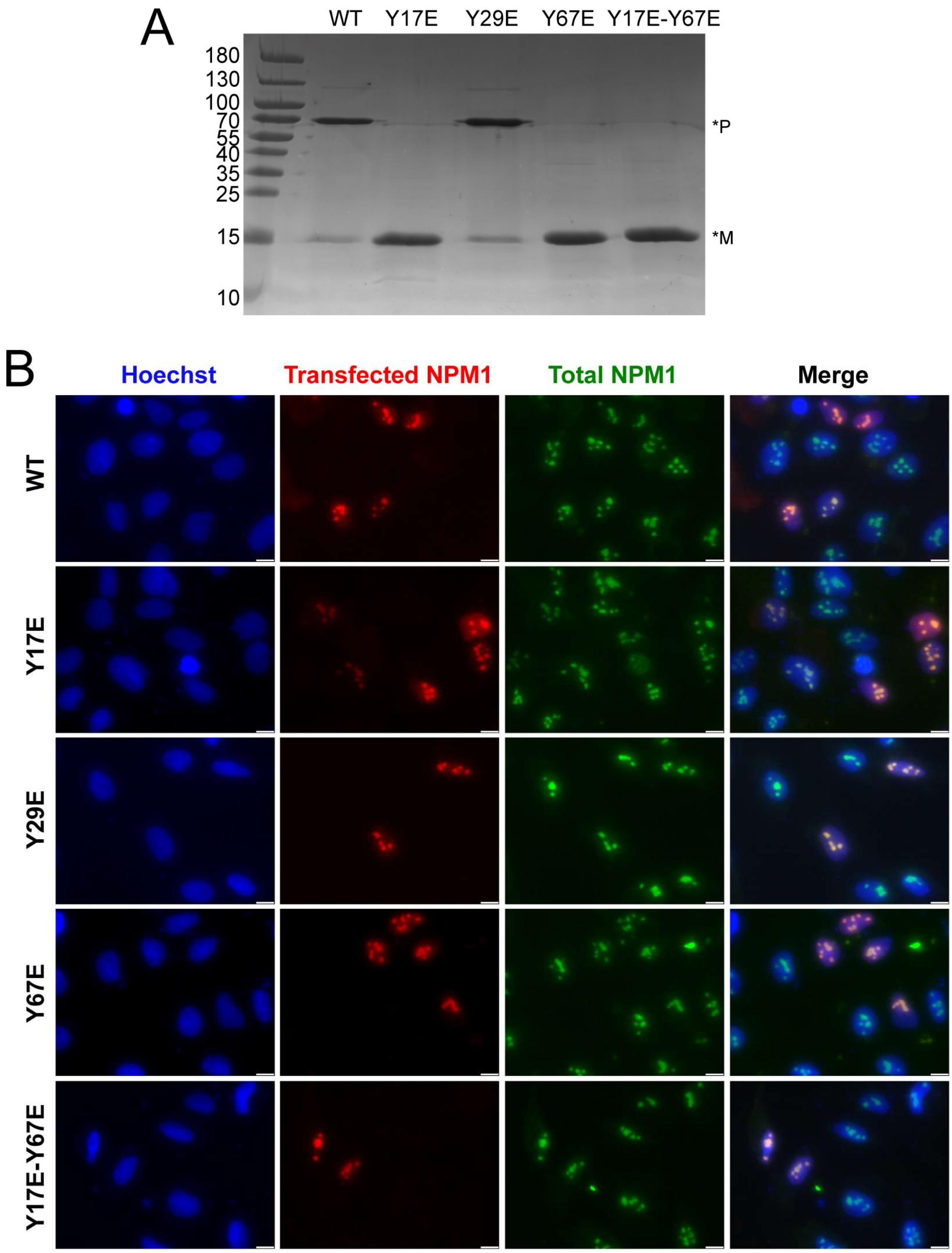

Figure S6. Characterization of the substitution by glutamate of tyrosines 17, 29 and 67 in NPM1. A) Gel electrophoresis analysis of the oligomerization equilibrium of Tyr-by-Glu NPM1$_{1\text{-}130}$ variants. The bands corresponding to the pentamer and monomer are indicated as *P or *M, respectively. **B)** Immunoflurescence microscopy images on U2OS cells of endogenous total NPM1 (green) and transfected *c*myc-tagged NPM1 Tyr-by-Glu phosphomimetic variants (red). The signal from transfected NPM1 variants was detected by an anti *c*myc-tag antibody. Scale bar = 10 µm.

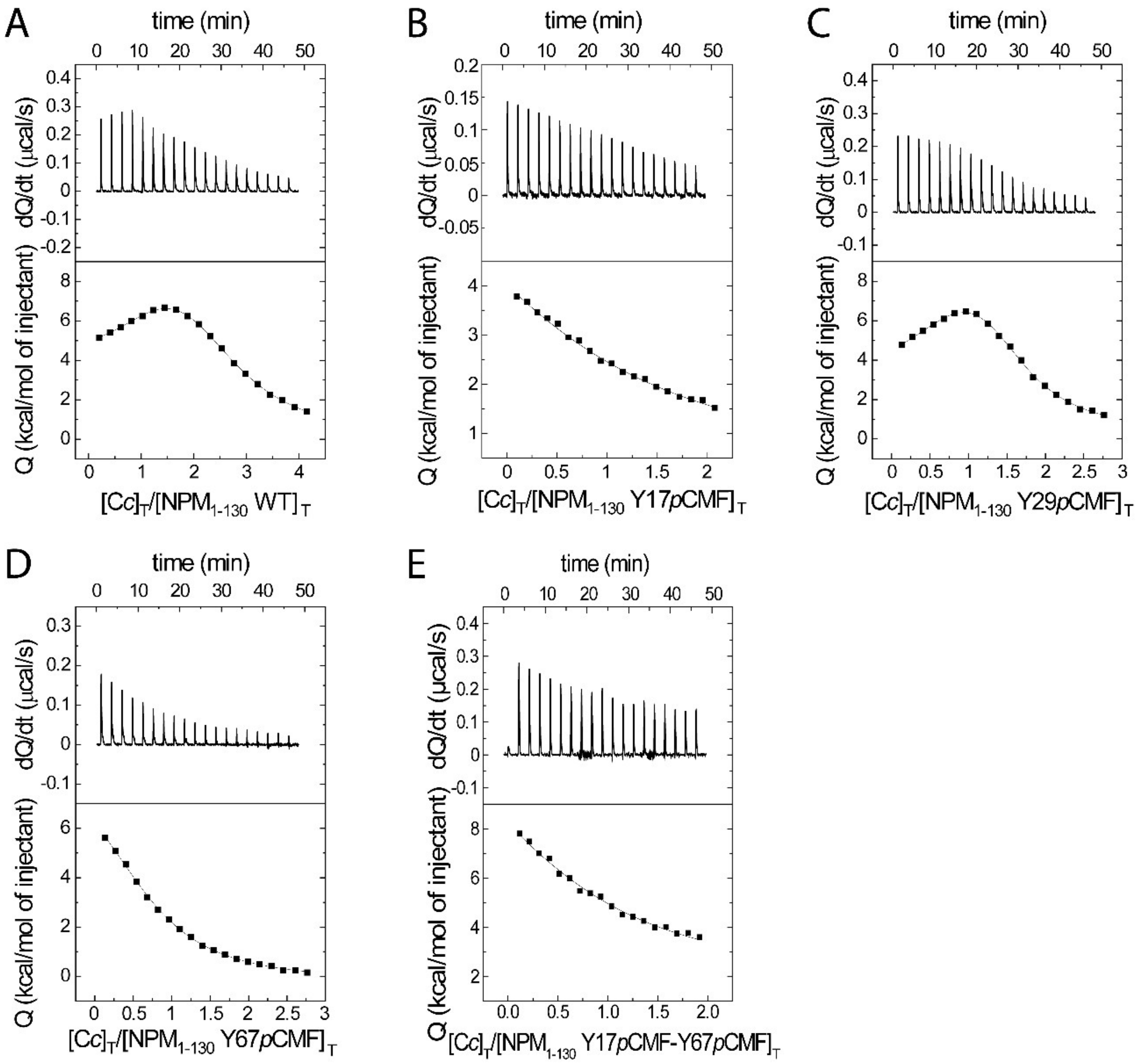

Figure S7. ITC assays for NPM1$_{1\text{-}130}$ variants with C*c*. Thermograms (*upper*) and binding isotherms (*lower*) calculated for each interaction are shown for WT **(A)**, Y17*p*CMF **(B)**, Y29*p*CMF **(C)**, Y67*p*CMF **(D)** and Y17*p*CMF-Y67*p*CMF **(E)** NPM1$_{1\text{-}130}$ variants.

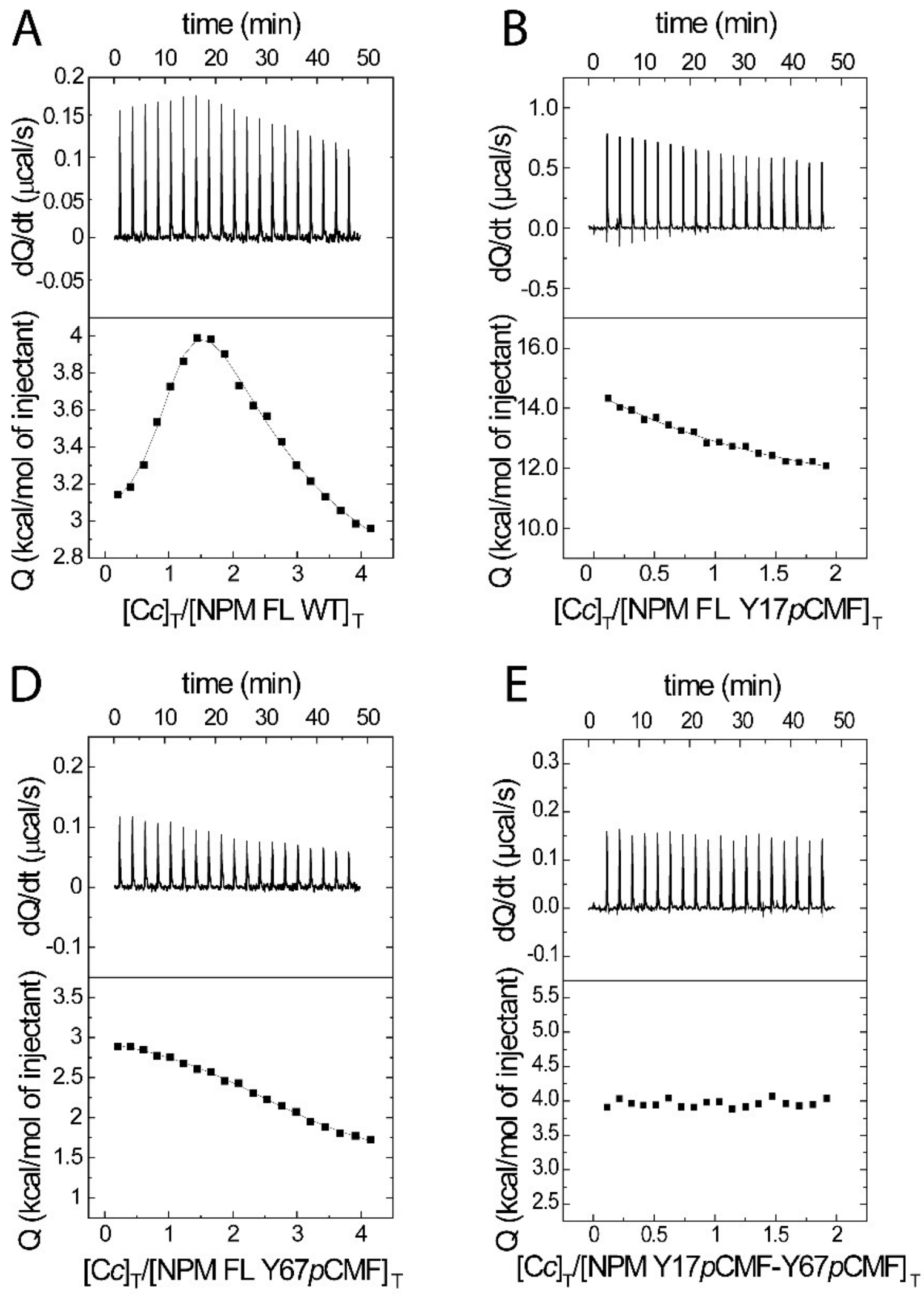

Figure S8. ITC assays for NPM1 FL variants with C*c*. Thermograms (*upper*) and binding isotherms (*lower*) calculated for each interaction are shown for WT **(A)**, Y17*p*CMF **(B)**, Y29*p*CMF **(C)**, Y67*p*CMF **(D)** and Y17*p*CMF-Y67*p*CMF **(E)** NPM1 FL variants.

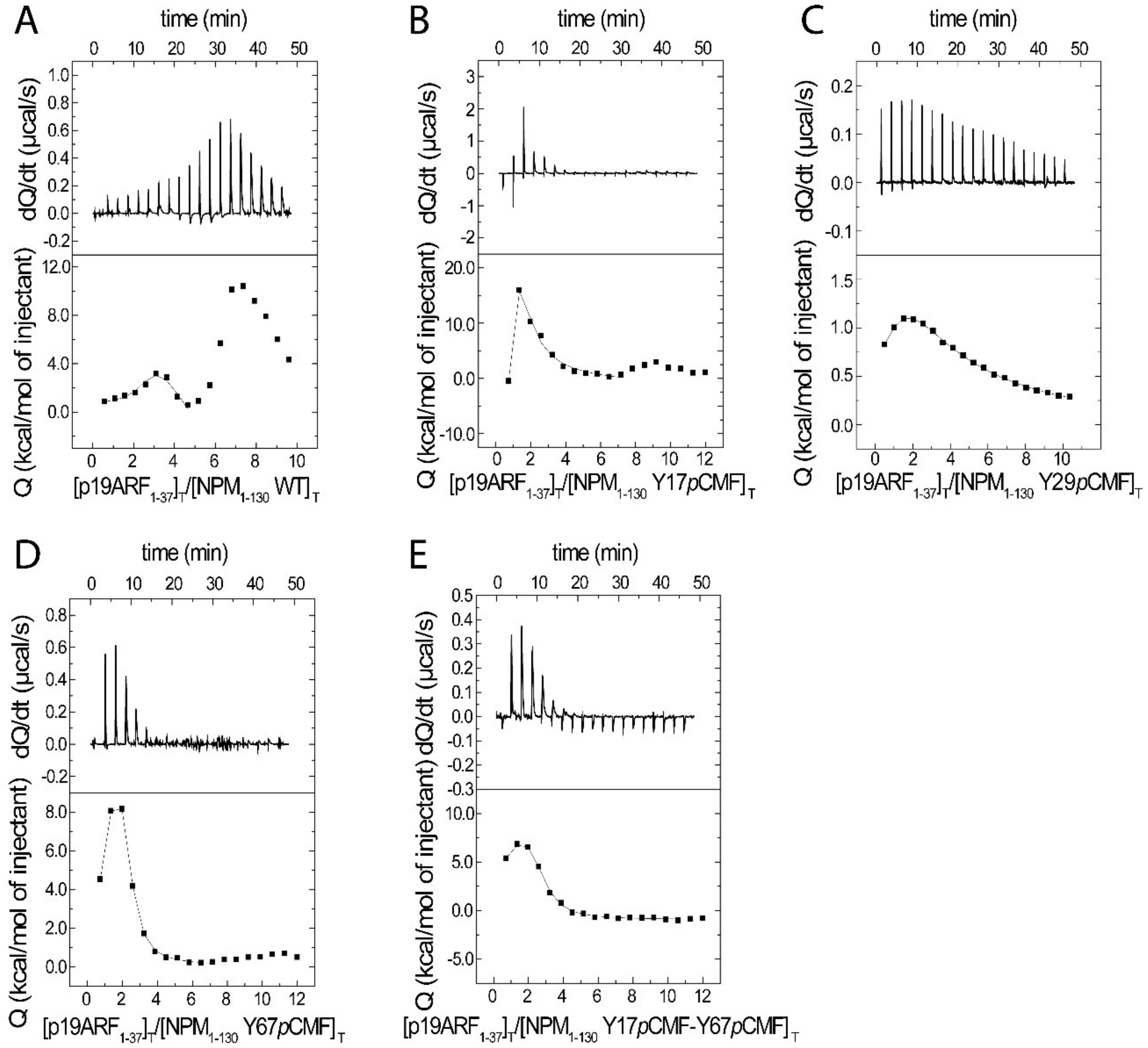

Figure S9. ITC assays for NPM1$_{1\text{-}130}$ variants with p19ARF. Thermograms (*upper*) and binding isotherms (*lower*) calculated for each interaction are shown for WT **(A)**, Y17*p*CMF **(B)**, Y29*p*CMF **(C)**, Y67*p*CMF **(D)** and Y17*p*CMF-Y67*p*CMF **(E)** NPM1$_{1\text{-}130}$ variants.

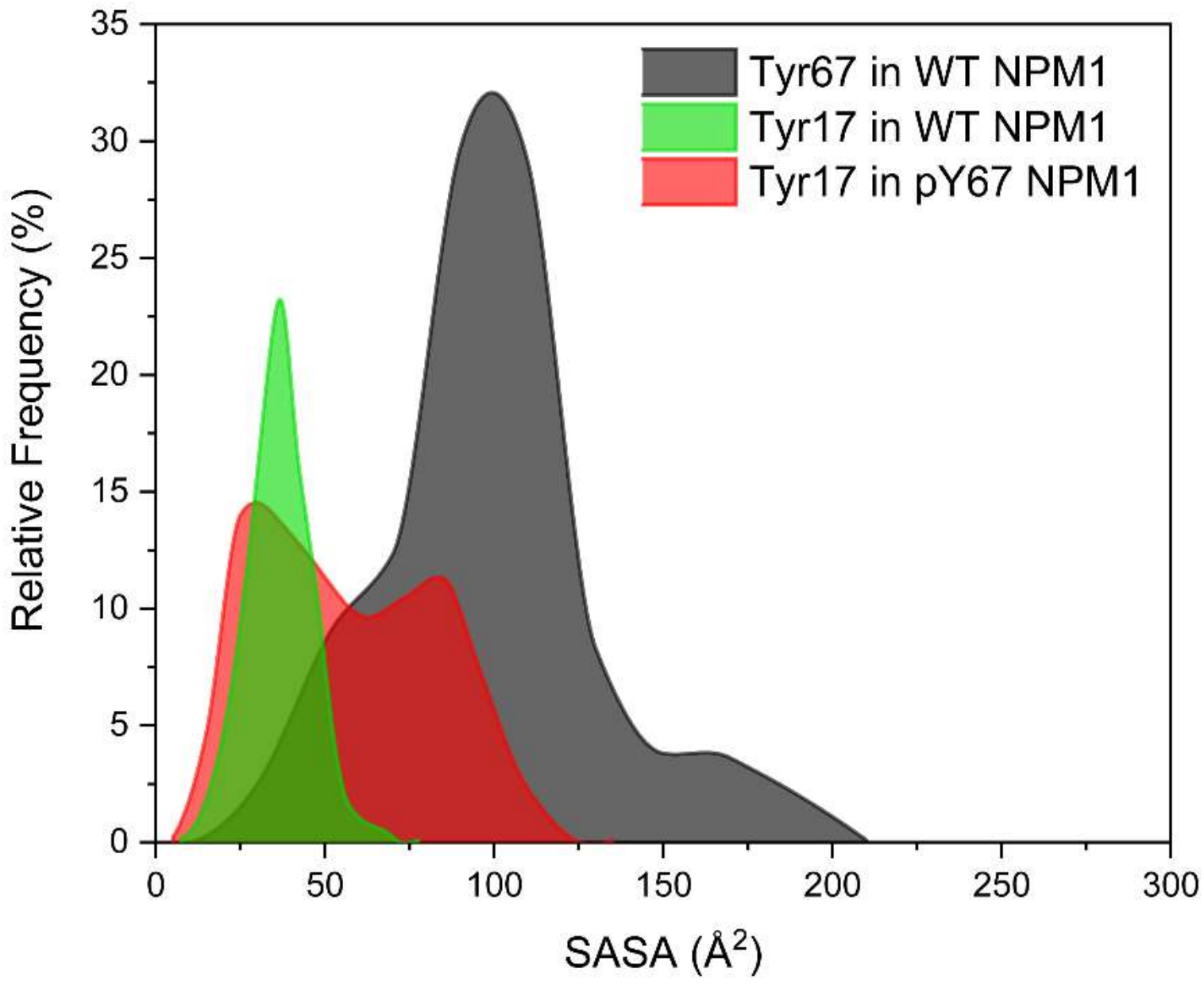

Figure S10. Solvent-accessible surface area (SASA) of residues 17 and 67 of NPM1$_{1\text{-}130}$ variants during the MDs. The relative frequency distribution is shown for Tyr67 in the NPM1$_{1\text{-}130}$ WT MD (gray) and for Tyr17 in the NPM1$_{1\text{-}130}$ WT MD (green) or in the NPM1$_{1\text{-}130}$ pY67 MD (red).

Table S1. FoldX prediction of the change in free energy (ΔΔG, kcal/mol) of phosphorylated NPM1$_{1\text{-}130}$ variants relative to the WT protein.

NPM1$_{1\text{-}130}$ Variant	ΔΔG (kcal/mol)
pY17	-0.6
pY29	-0.4
pY67	6.5
pY17 - pY29	-0.9
pY17 - pY67	12.6
pY29 - pY67	7.6
pY17 - pY29 - pY67	13.0

Table S2. Plasmids used in this work.

Plasmid	Description	Reference/Source
pEVOL-*p*CMF	pEVOL carrying specific aminoacyl-tRNA synthetase for *p*CMF	55
pEt28a(+) NPM1 FL	Bacterial expression vector for NPM1 FL	12
pEt28a(+) NPM1 FL Y17Amber	Bacterial expression vector for NPM1 FL Y17Amber	This study
pEt28a(+) NPM1 FL Y29Amber	Bacterial expression vector for NPM1 FL Y29Amber	This study
pEt28a(+) NPM1 FL Y67Amber	Bacterial expression vector for NPM1 FL Y67Amber	This study
pEt28a(+) NPM1 FL Y17Amber-Y67Amber	Bacterial expression vector for NPM1 FL Y17Amber-Y67Amber	This study
pEt28a(+) $NPM1_{1\text{-}130}$	Bacterial expression vector for $NPM1_{1\text{-}130}$	12
pEt28a(+) $NPM1_{1\text{-}130}$ Y17Amber	Bacterial expression vector for $NPM1_{1\text{-}130}$ Y17Amber	This study
pEt28a(+) $NPM1_{1\text{-}130}$ Y29Amber	Bacterial expression vector for $NPM1_{1\text{-}130}$ Y29Amber	This study
pEt28a(+) $NPM1_{1\text{-}130}$ Y67Amber	Bacterial expression vector for $NPM1_{1\text{-}130}$ Y67Amber	This study
pEt28a(+) $NPM1_{1\text{-}130}$ Y17Amber-Y67Amber	Bacterial expression vector for $NPM1_{1\text{-}130}$ Y17Amber-Y67Amber	This study
pEt28a(+) $NPM1_{1\text{-}130}$ Y17E	Bacterial expression vector for $NPM1_{1\text{-}130}$ Y17E	This study
pEt28a(+) $NPM1_{1\text{-}130}$ Y29E	Bacterial expression vector for $NPM1_{1\text{-}130}$ Y29E	This study
pEt28a(+) $NPM1_{1\text{-}130}$ Y67E	Bacterial expression vector for $NPM1_{1\text{-}130}$ Y67E	This study
pEt28a(+) $NPM1_{1\text{-}130}$ Y17E-Y67E	Bacterial expression vector for $NPM1_{1\text{-}130}$ Y17E-Y67E	This study
pcDNA3.1 NPM1-cmyc	Mammalian expression vector for cmyc-tagged NPM1	This study
pcDNA3.1 NPM1-cmyc Y17F	Mammalian expression vector for cmyc-tagged NPM1 Y17F	This study
pcDNA3.1 NPM1-cmyc Y29F	Mammalian expression vector for cmyc-tagged NPM1 Y29F	This study
pcDNA3.1 NPM1-cmyc Y67F	Mammalian expression vector for cmyc-tagged NPM1 Y67F	This study
pcDNA3.1 NPM1-cmyc Y17F-Y67F	Mammalian expression vector for cmyc-tagged NPM1 Y17F-Y67F	This study
pcDNA3.1 NPM1-cmyc Y17E	Mammalian expression vector for cmyc-tagged NPM1 Y17E	This study
pcDNA3.1 NPM1-cmyc Y29E	Mammalian expression vector for cmyc-tagged NPM1 Y29E	This study
pcDNA3.1 NPM1-cmyc Y67E	Mammalian expression vector for cmyc-tagged NPM1 Y67E	This study
pcDNA3.1 NPM1-cmyc Y17E-Y67E	Mammalian expression vector for cmyc-tagged NPM1 Y17E-Y67E	This study

Table S3. Oligonucleotides used in this work.

Oligonucleotide	Sequence (5'-3')
NPM1-Y17Amber-Fw	AACTTTCTTTTCGGTTGTGAACTAAAGGCCGAC
NPM1-Y17Amber-Rv	AAGAAAGTTCTGGGGCCTCAGGGGG
NPM1-Y29Amber-Fw	GATTATCACTTTAAGGTGGATAATGATGAAAATGA
NPM1-Y29Amber-Rv	GTGATAATCTTTGTCGGCCTTTAGTTCACA
NPM1-Y67Amber-Fw	AATTTCGAAGGCAGTCCAATTAAAGTAACACTG
NPM1-Y67Amber-Rv	TTCGAAATTCATTGCCTCTGCTTCAACAATG
NPM1-Y17F-Fw	AACTTTCTTTTCGGTTGTGAACTAAAGGCCGAC
NPM1-Y17F-Rv	AAGAAAGTTCTGGGGCCTCAGGGGG
NPM1-Y29F-Fw	GATTATCACTTTAAGGTGGATAATGATGAAAATGA
NPM1-Y29F-Rv	GTGATAATCTTTGTCGGCCTTTAGTTCACA
NPM1-Y67F-Fw	AATTTCGAAGGCAGTCCAATTAAAGTAACACTG
NPM1-Y67F-Rv	TTCGAAATTCATTGCCTCTGCTTCAACAATG
NPM1-Y17E-Fw	AACGAACTTTTCGGTTGTGAACTAAAGGCCGAC
NPM1-Y17E-Rv	AAGTTCGTTCTGGGGCCTCAGGGGGC
NPM1-Y29E-Fw	GATGAGCACTTTAAGGTGGATAATGATGAAAA
NPM1-Y29E-Rv	AGTGCTCATCTTTGTCGGCCTTTAGTTC
NPM1-Y67E-Fw	GGCAATGAATGAAGAAGGCAGTCCAATTAAAGTAACACTGGC
NPM1-Y67E-Rv	GACTGCCTTCTTCATTCATTGCCTCTGCTTCAACAATGTGC
